# Supplementary material for: Flexible ferroelectric organic crystals
Source: Nat Commun. 2016 Oct 13;7:13108. doi: 10.1038/ncomms13108 (PMC5065626; doi:10.1038/ncomms13108)
Supplement: Supplementary Information — Supplementary Figures 1-21, Supplementary Tables 1-17, Supplementary Notes 1-4, Supplementary Methods and Supplementary References [file ncomms13108-s1.pdf]

Supplementary Figures

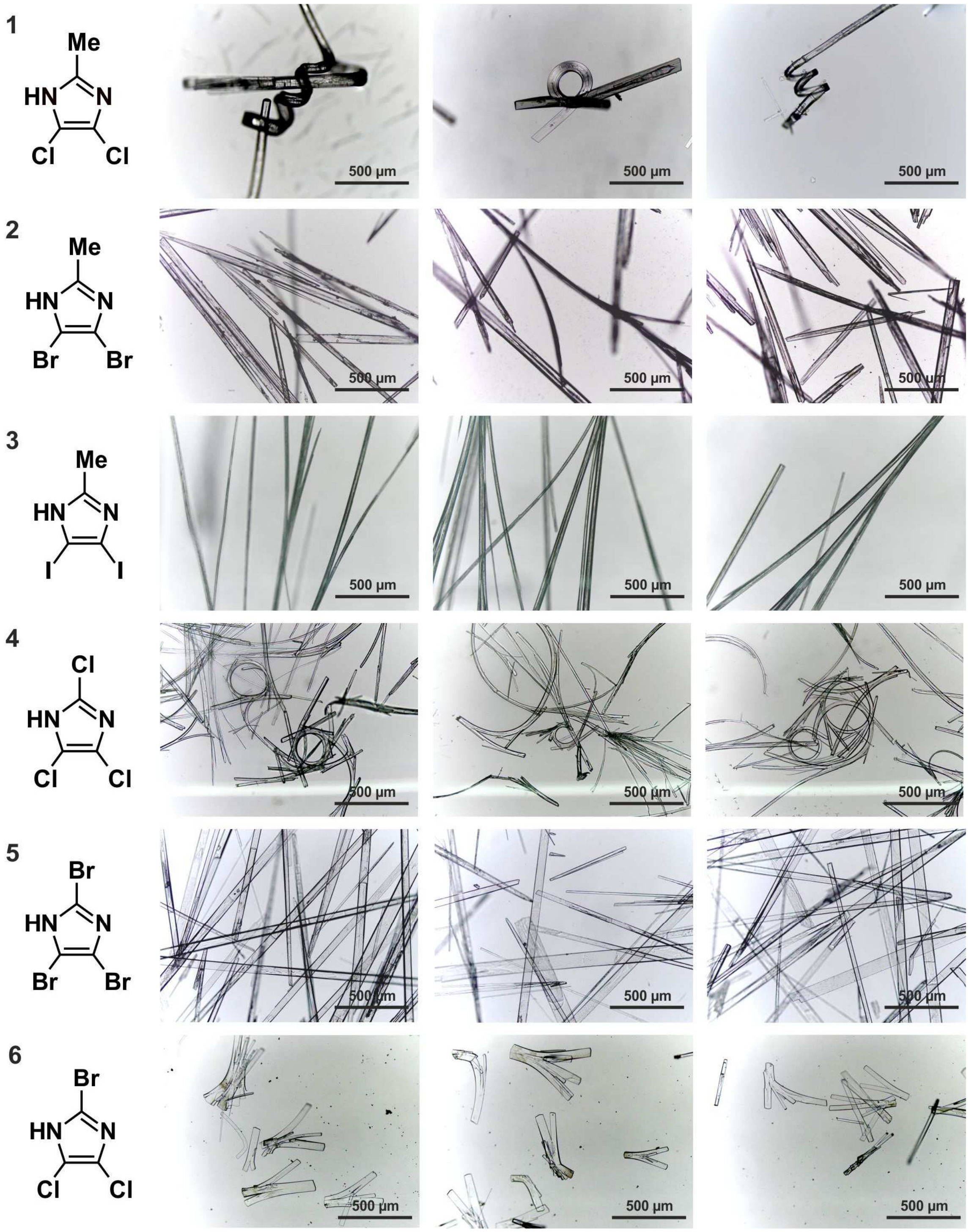

Supplementary Figure 1. Optical microscopy images.

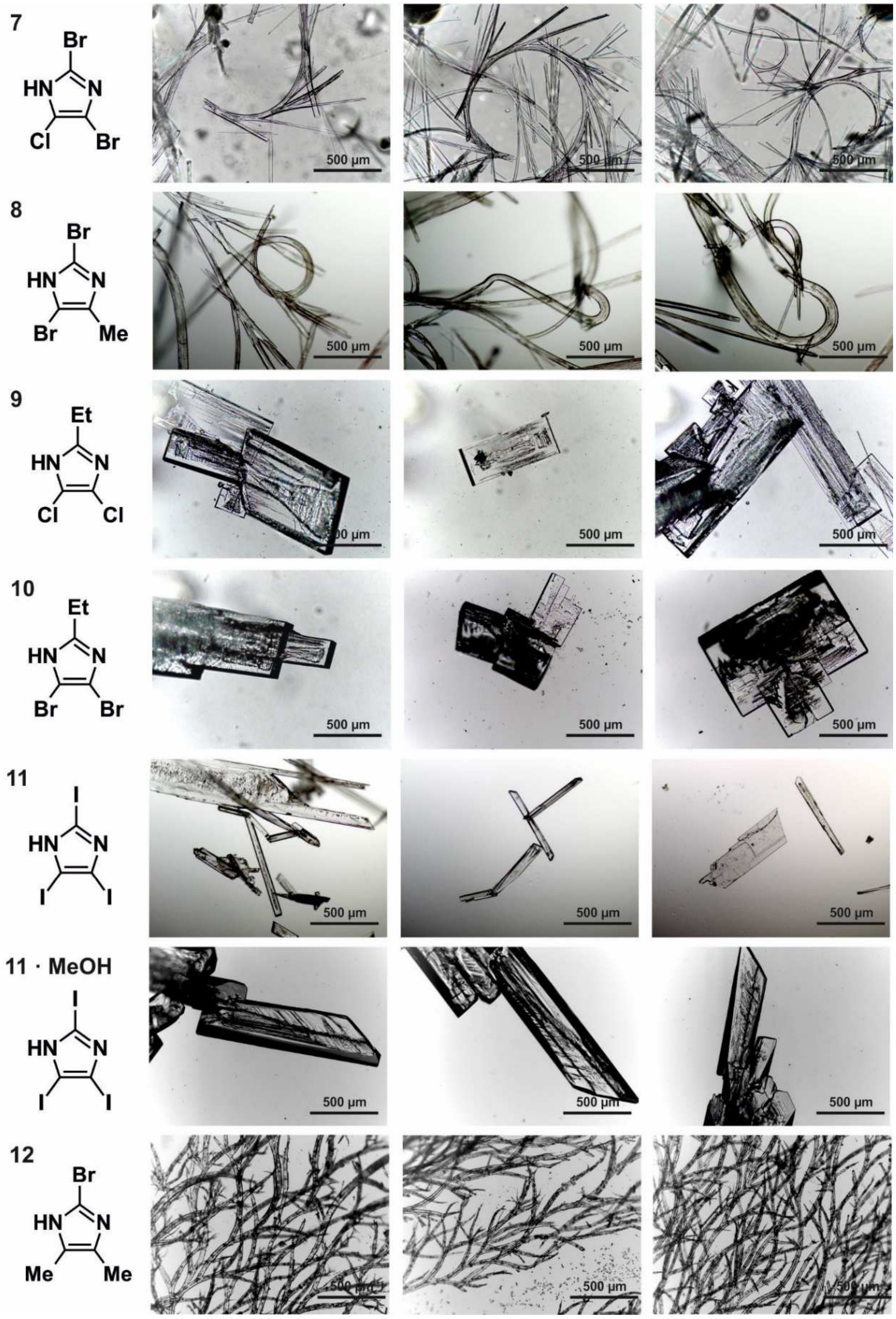

Supplementary Figure 1 cont. Optical microscopy images.

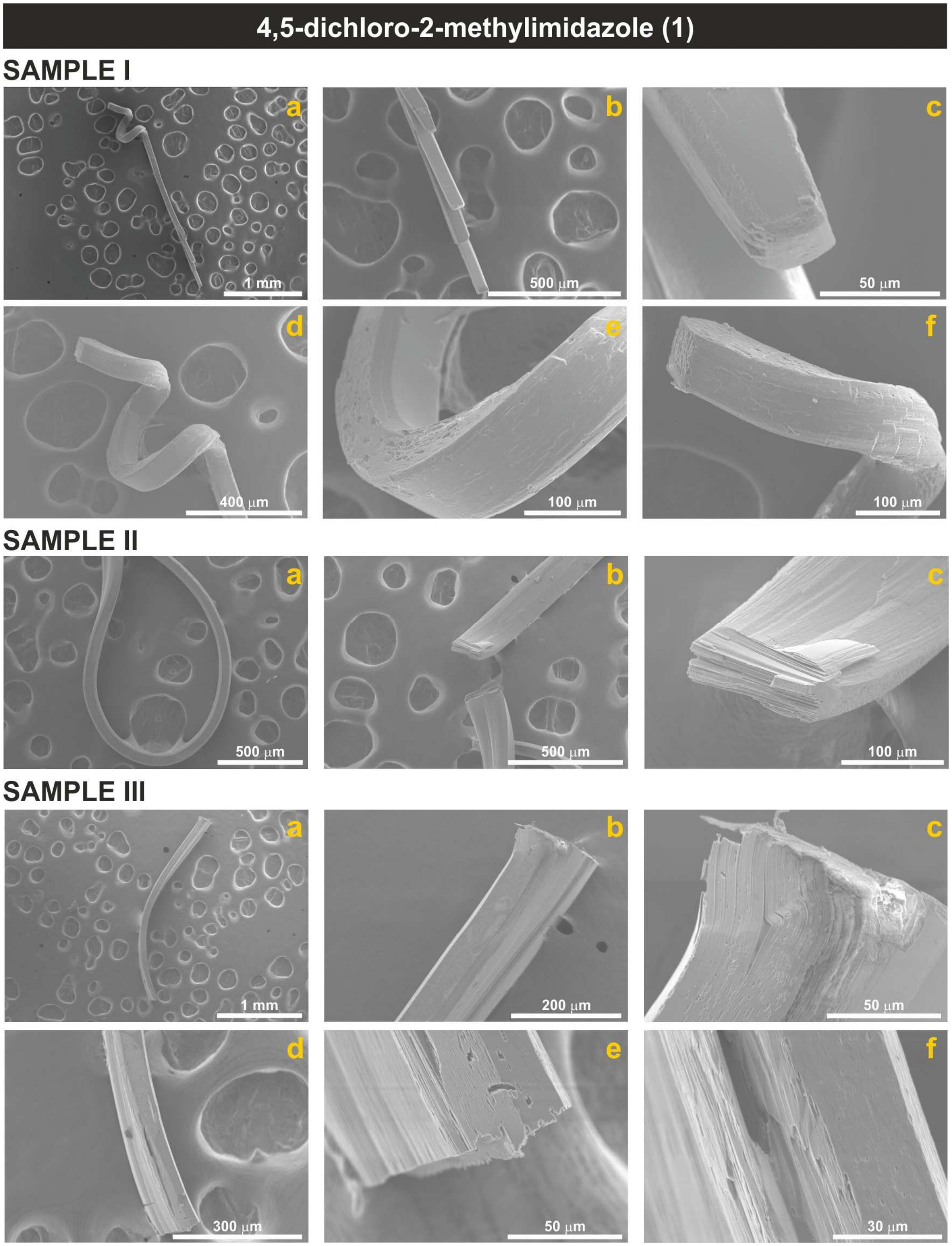

Supplementary Figure 2. SEM Images of 4,5-dichloro-2-methylimidazole (**1**).

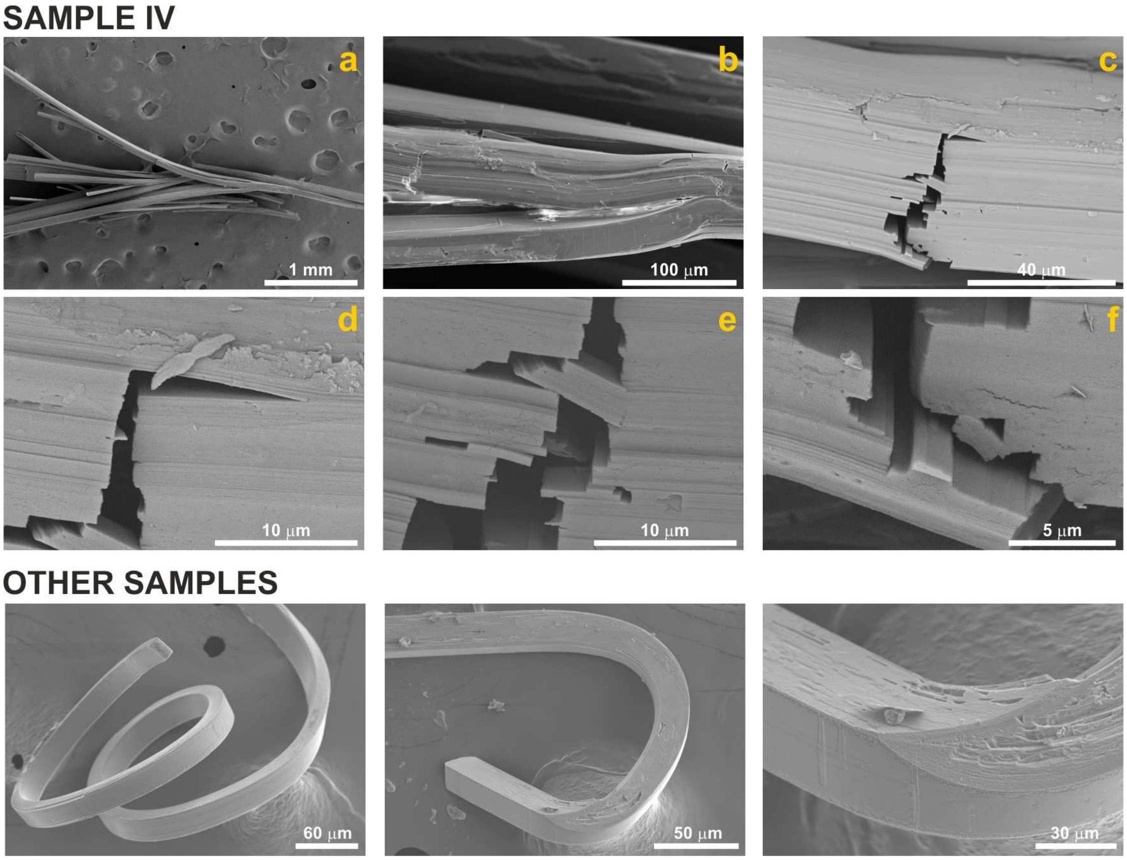

Supplementary Figure 2 cont. SEM Images of 4,5-dichloro-2-methylimidazole (**1**).

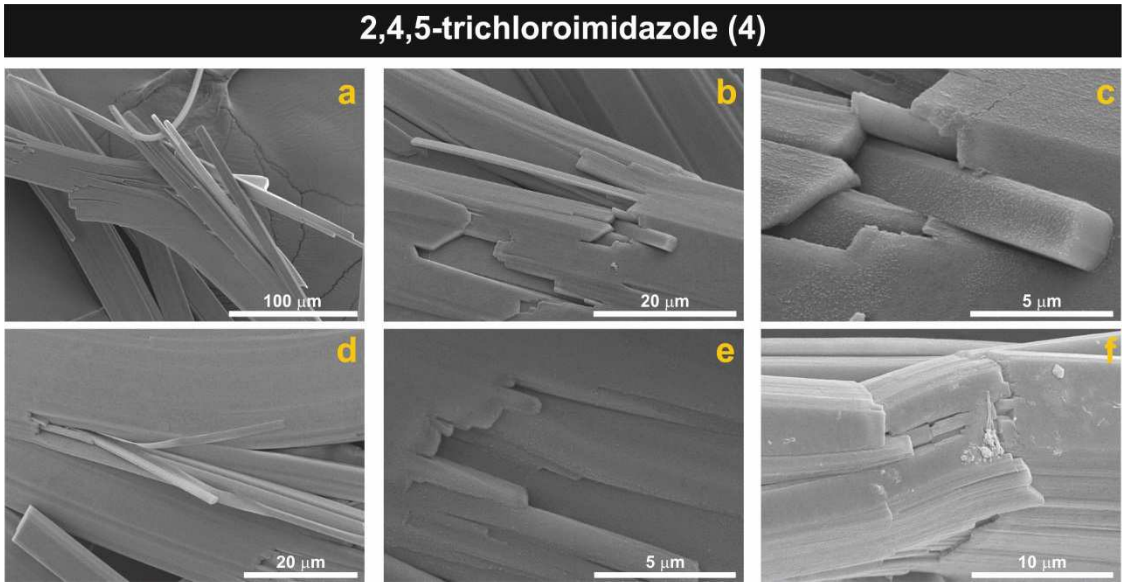

Supplementary Figure 3. SEM Images of 2,4,5-trichloroimidazole (**4**).

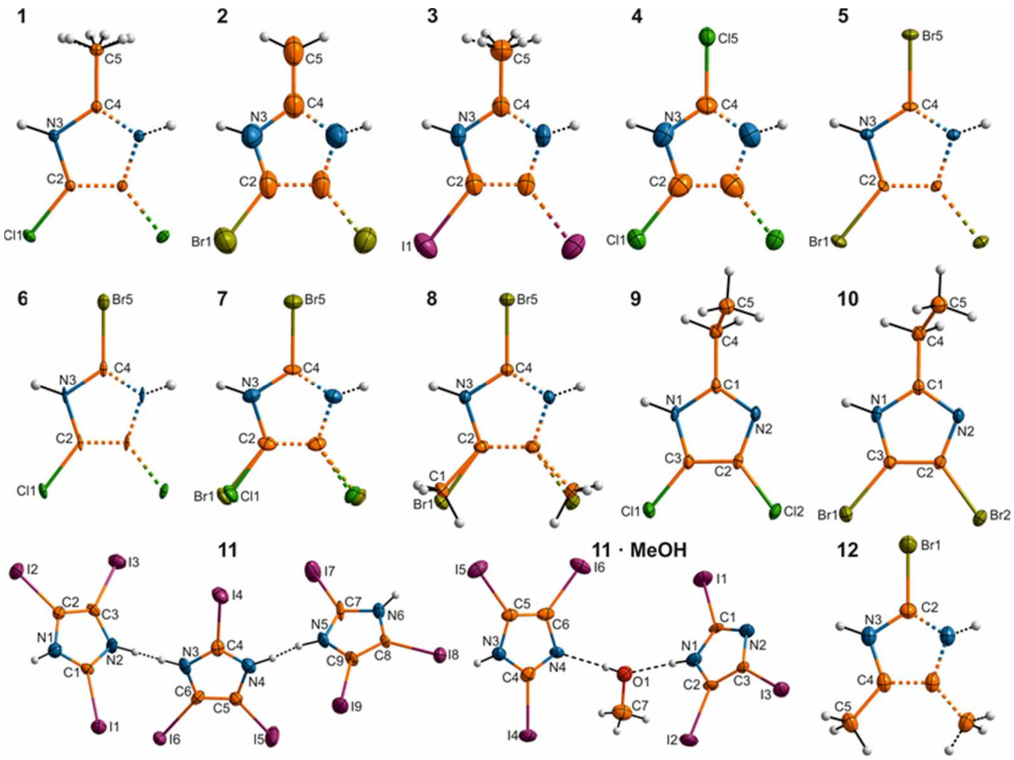

Supplementary Figure 4. Single-crystal X-ray diffraction. The asymmetric parts of the unit cell of 4,5-dichloro-2-methylimidazole (**1**), 4,5-dibromo-2-methylimidazole (**2**, reported), 4,5-diiodo-2-methylimidazole (**3**), 2,4,5-trichloroimidazole (**4**), 2,4,5-tribromoimidazole (**5**), 2-bromo-4,5-dichloroimidazole (**6**), 2,4-dibromo-5-chloroimidazole (**7**), 2,4-dibromo-5-methylimidazole (**8**), 4,5-dichloro-2-ethylimidazole (**9**), 4,5-dibromo-2-ethylimidazole (**10**), 2,4,5-triiodoimidazole (**11**), 2,4,5-triiodoimidazole · MeOH (**11 · MeOH**), and 2-bromo-4,5-dimethylimid-azole (**12**, reported) marked with solid lines and symmetrical equivalents marked with dashed lines. The hydrogen-bonding interactions are depicted as black dashed lines. Thermal ellipsoid are shown at 50% probability.

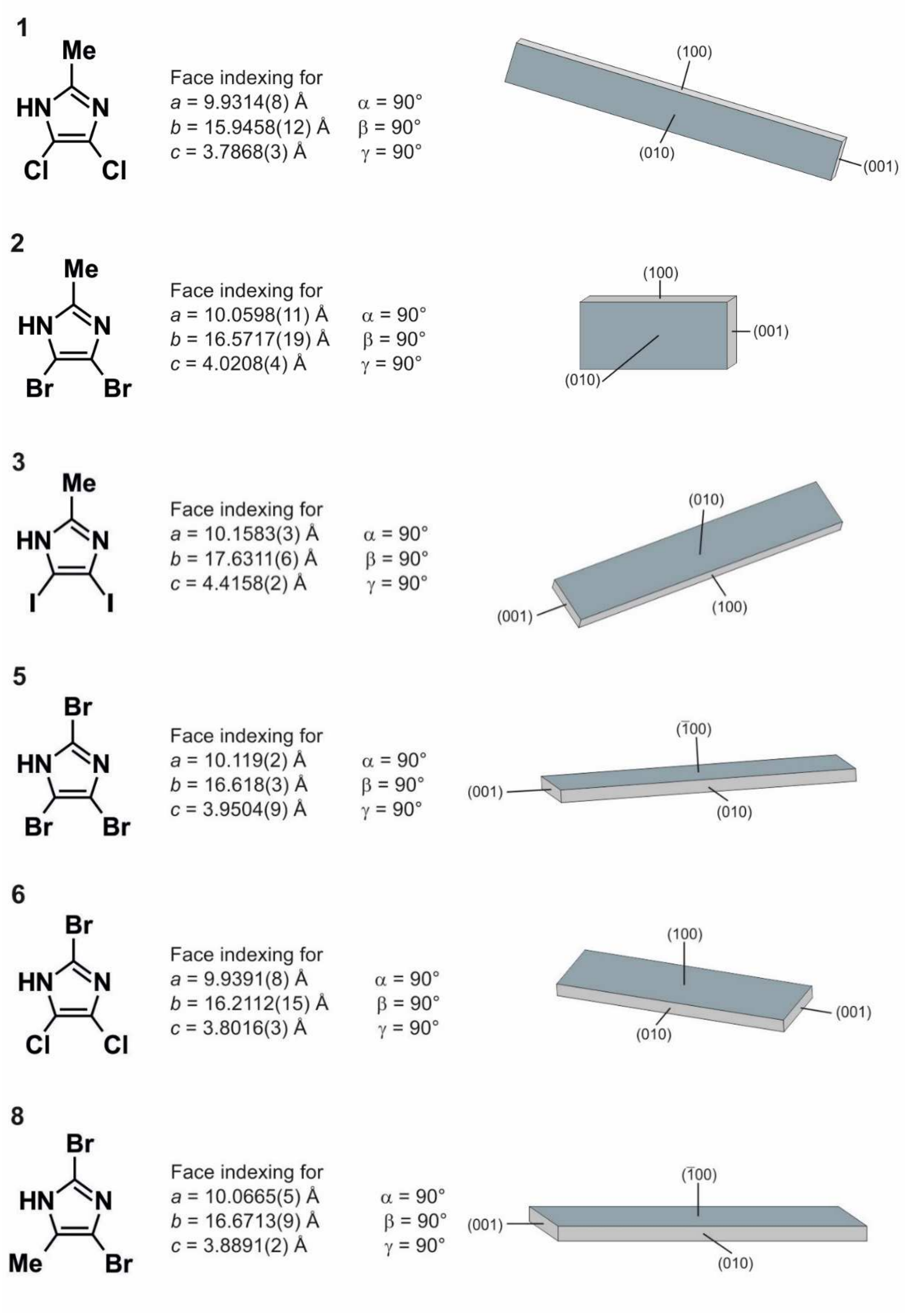

Supplementary Figure 5. Crystal orientation of selected compounds.

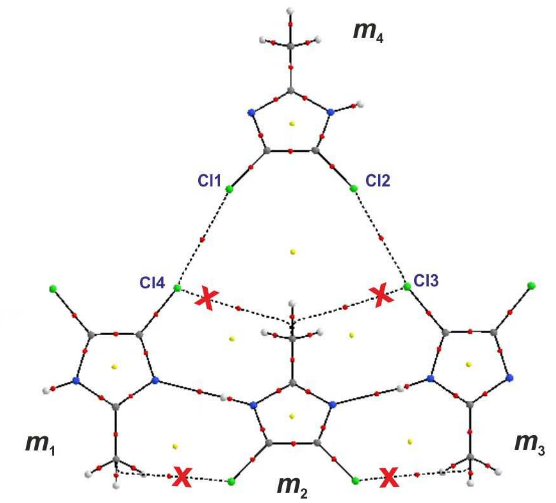

Supplementary Figure 6. Theoretical calculations. Structural motifs and bond paths obtained from AIM analyses of **1**. Small circles are attributed to the critical points: red, bond critical point; yellow, ring critical point.

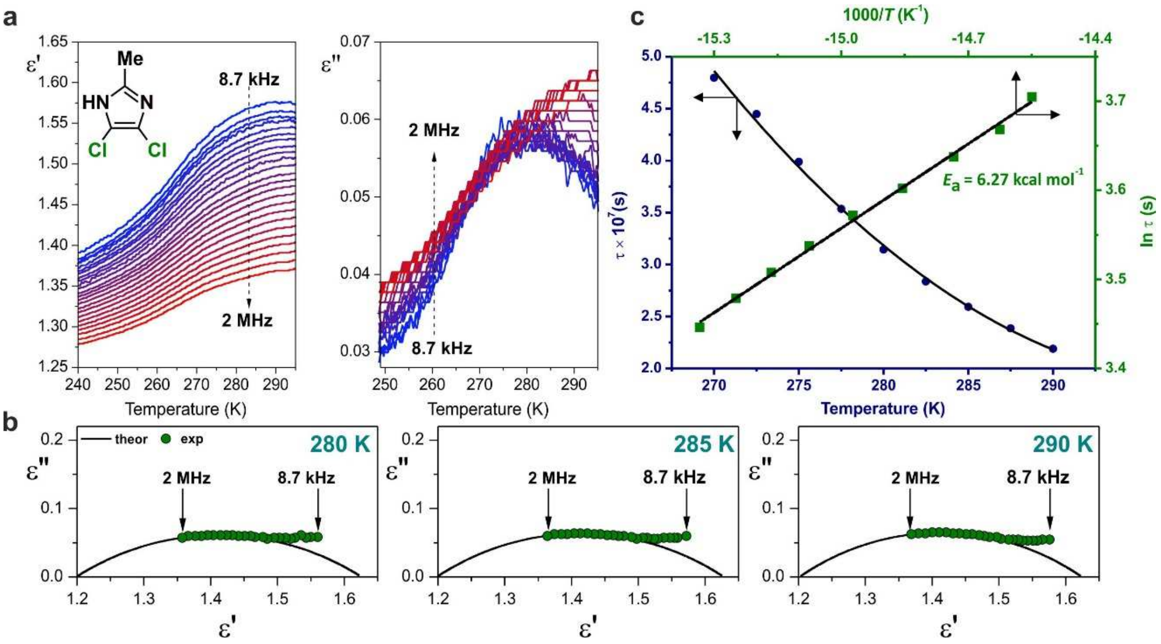

Supplementary Figure 7. Dielectric spectroscopy of 1. a) Temperature dependence of the real and imaginary parts of the complex electric permittivity obtained for a 4,5-dichloro-2-methylimidazole (**1**) pellet on heating. The colour gradient depicts the change in frequency from 8.7 kHz (blue) to 2 MHz (red). b) Cole-Cole plots of ε'' versus ε' at selected temperatures. c) Plots of relaxation times τ versus temperature and lnτ versus reciprocal temperature. The calculated activation energy E_a is given.

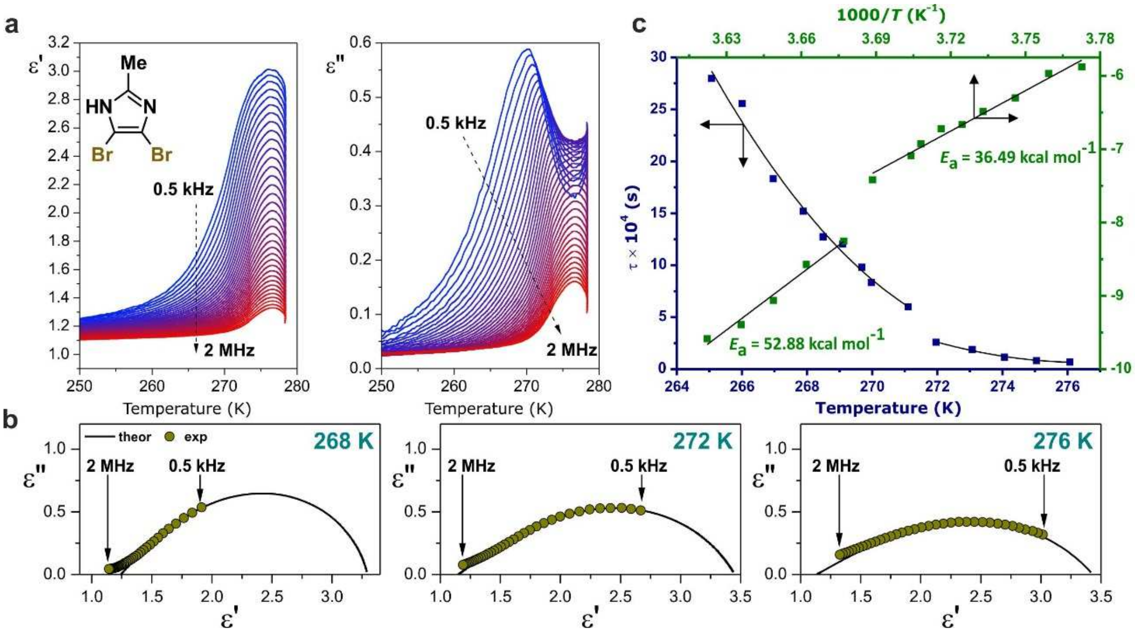

Supplementary Figure 8. Dielectric spectroscopy of 2. a) Temperature dependence of the real and imaginary parts of the complex electric permittivity obtained for a 4,5-dibromo-2-methylimidazole (**2**) pellet on cooling. The colour gradient depicts the change in frequency from 0.5 kHz (blue) to 2 MHz (red). b) Havriliak-Negami plots of ε'' versus ε' at selected temperatures. c) Plots of relaxation times τ versus temperature and lnτ versus reciprocal temperature. The calculated activation energy values are given.

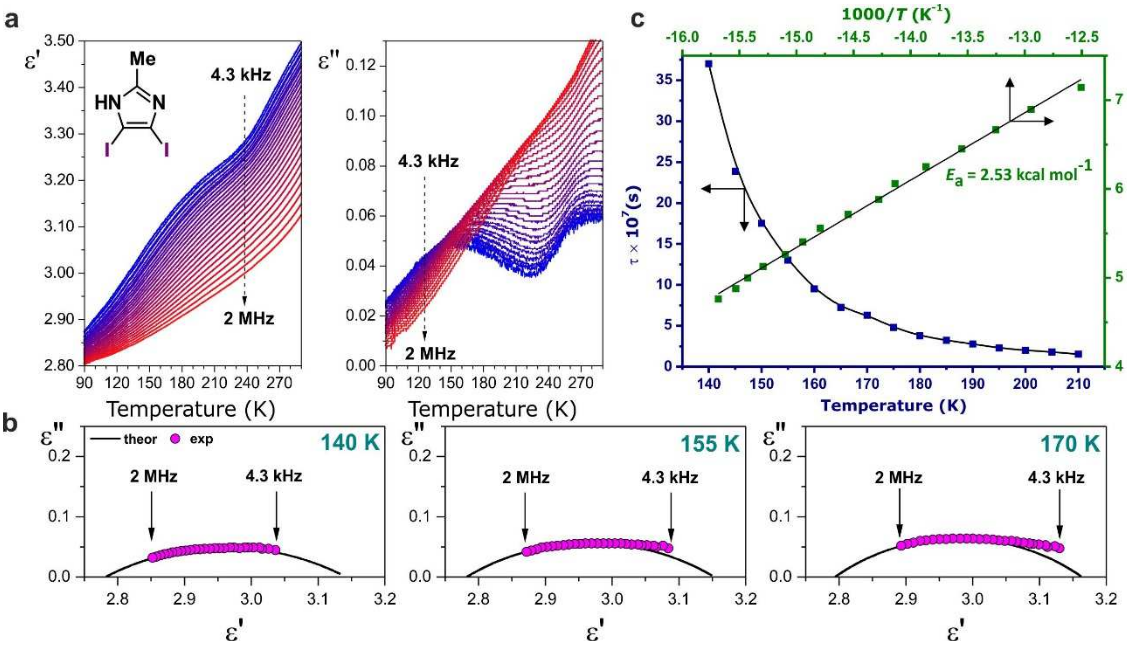

Supplementary Figure 9. Dielectric spectroscopy of 3. a) Temperature dependence of the real and imaginary parts of the complex electric permittivity obtained for a 4,5-diiodo-2-methylimidazole (**3**) pellet on cooling. The colour gradient depicts the change in frequency from 4.3 kHz (blue) to 2 MHz (red). b) Cole-Cole plots of ε'' versus ε' at selected temperatures. c) Plots of relaxation times τ versus temperature and lnτ versus reciprocal temperature. The calculated activation energy E_a is given.

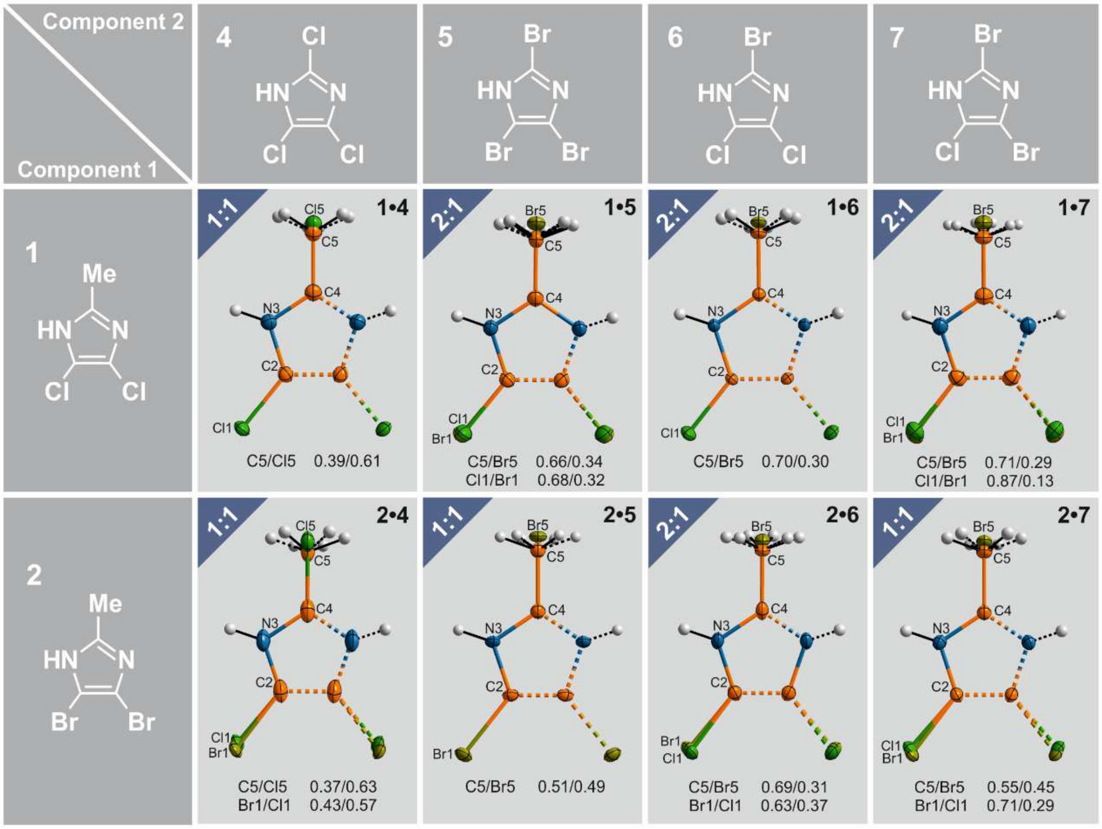

Supplementary Figure 10. Single-crystal X-ray diffraction of mixed crystals. Asymmetric units of **1•4**, **1•5**, **1•6**, **1•7**, **2•4**, **2•5**, **2•6**, and **2•7** solid solutions marked with solid lines and symmetrical equivalents marked with dotted lines. Ratios in which components **1** and **2** were mixed are presented and occupancy factors of disordered atoms are given. Thermal ellipsoids are shown at 50% probability. Due to the overlap of positions of disordered Cl1 and Br1 atoms, error in their occupation factors might be higher than for the disordered C5 and Cl5/Br5 atoms.

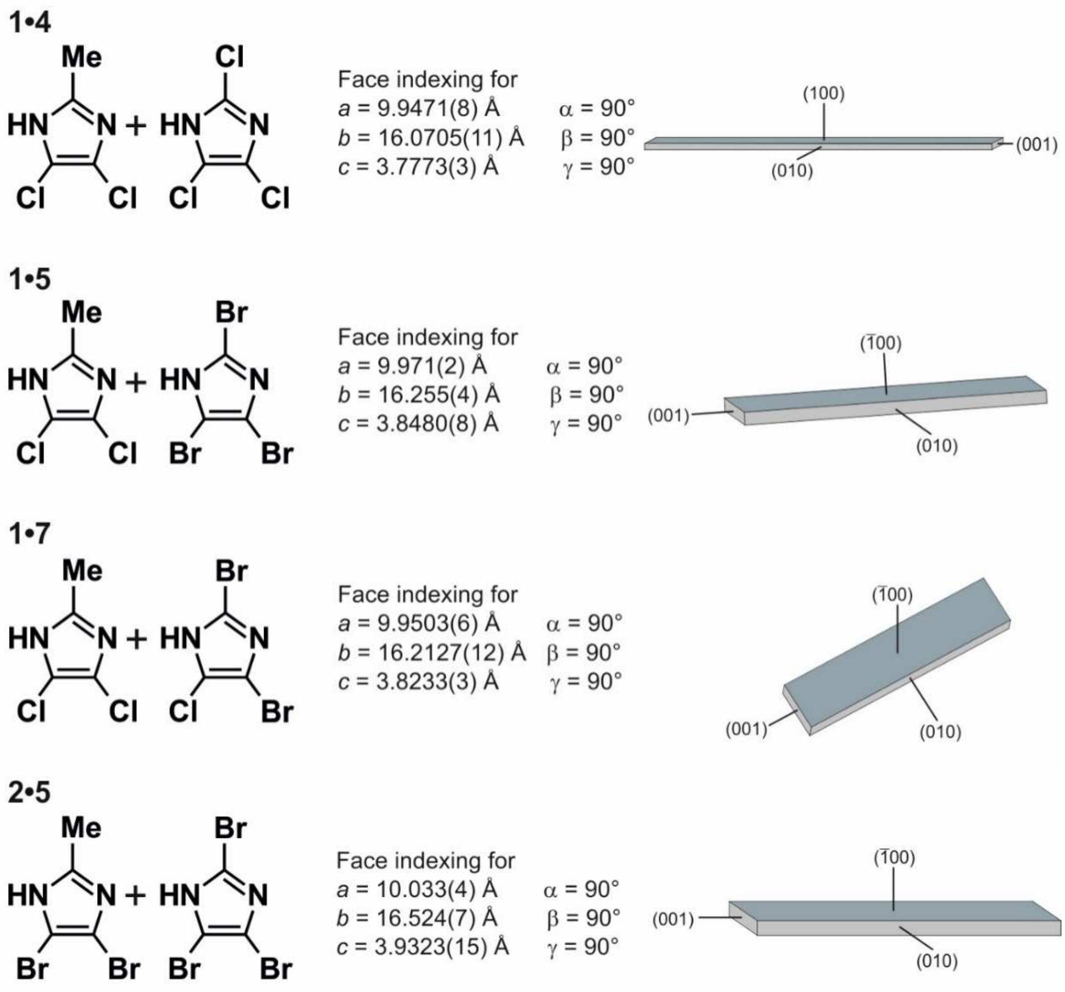

Supplementary Figure 11. Crystal orientation of selected mixed crystals.

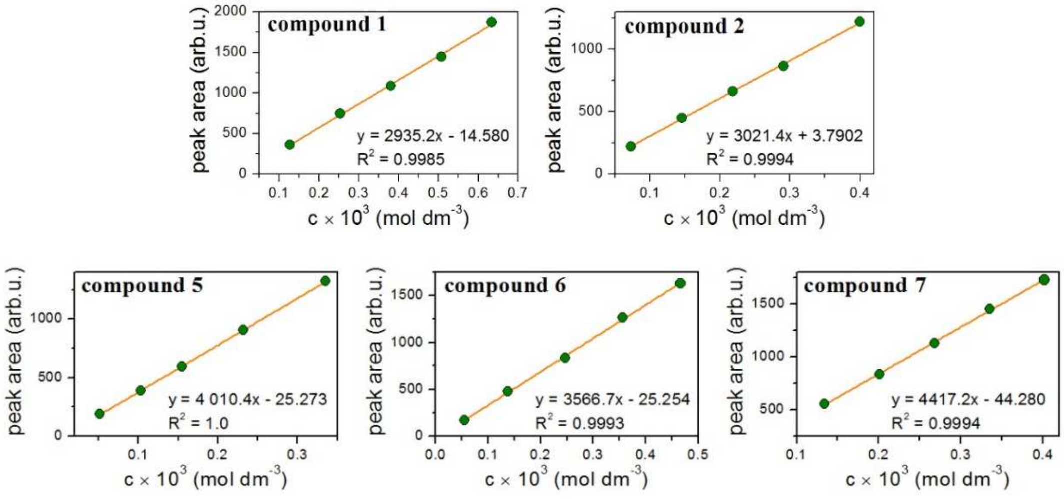

Supplementary Figure 12. HPLC experiments. Peak area *vs* concentration calibration lines for compounds **1**, **2**, and **5**–**7** obtained using HPLC.

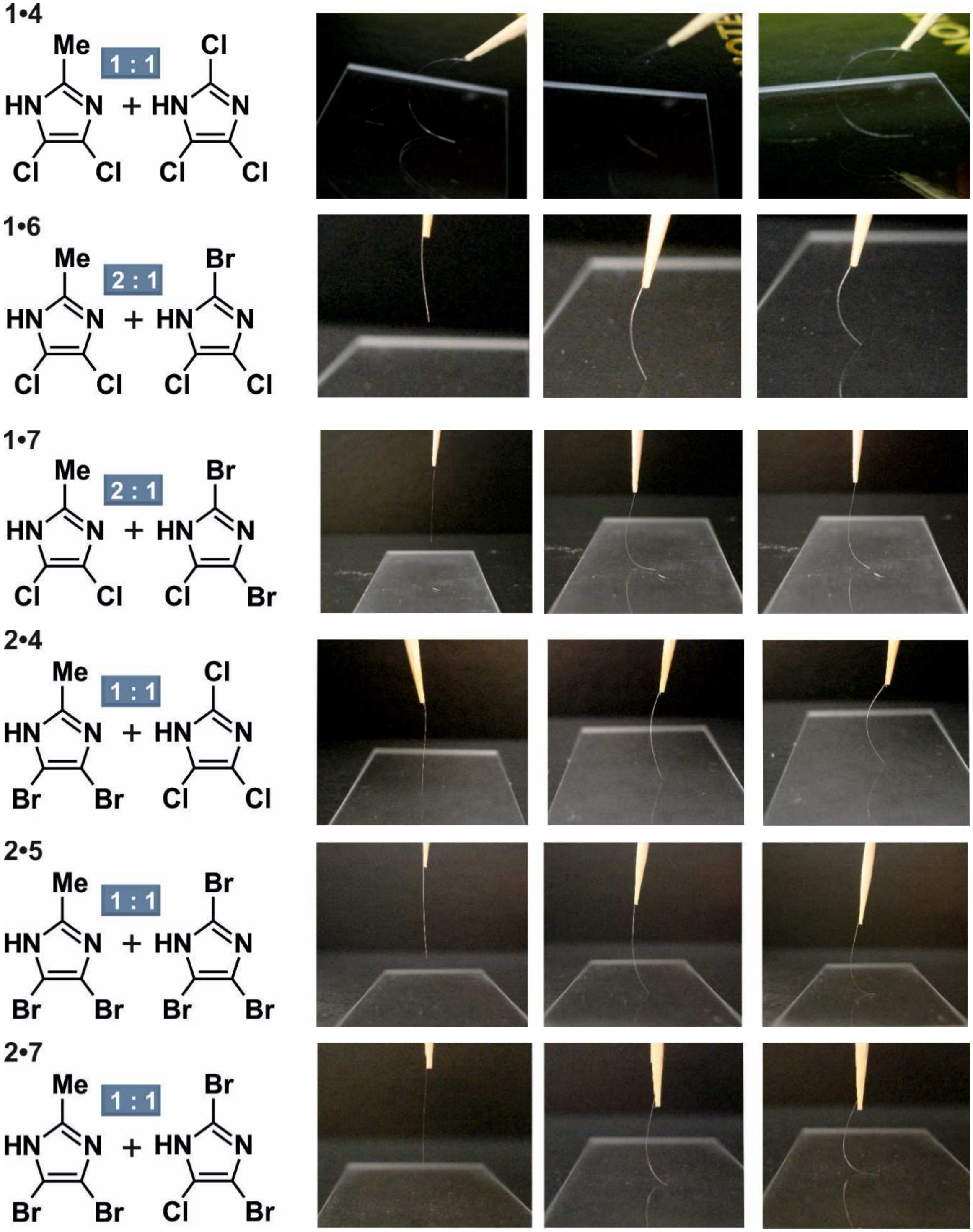

Supplementary Figure 13. Mechanical properties of mixed haloimidazole crystals.

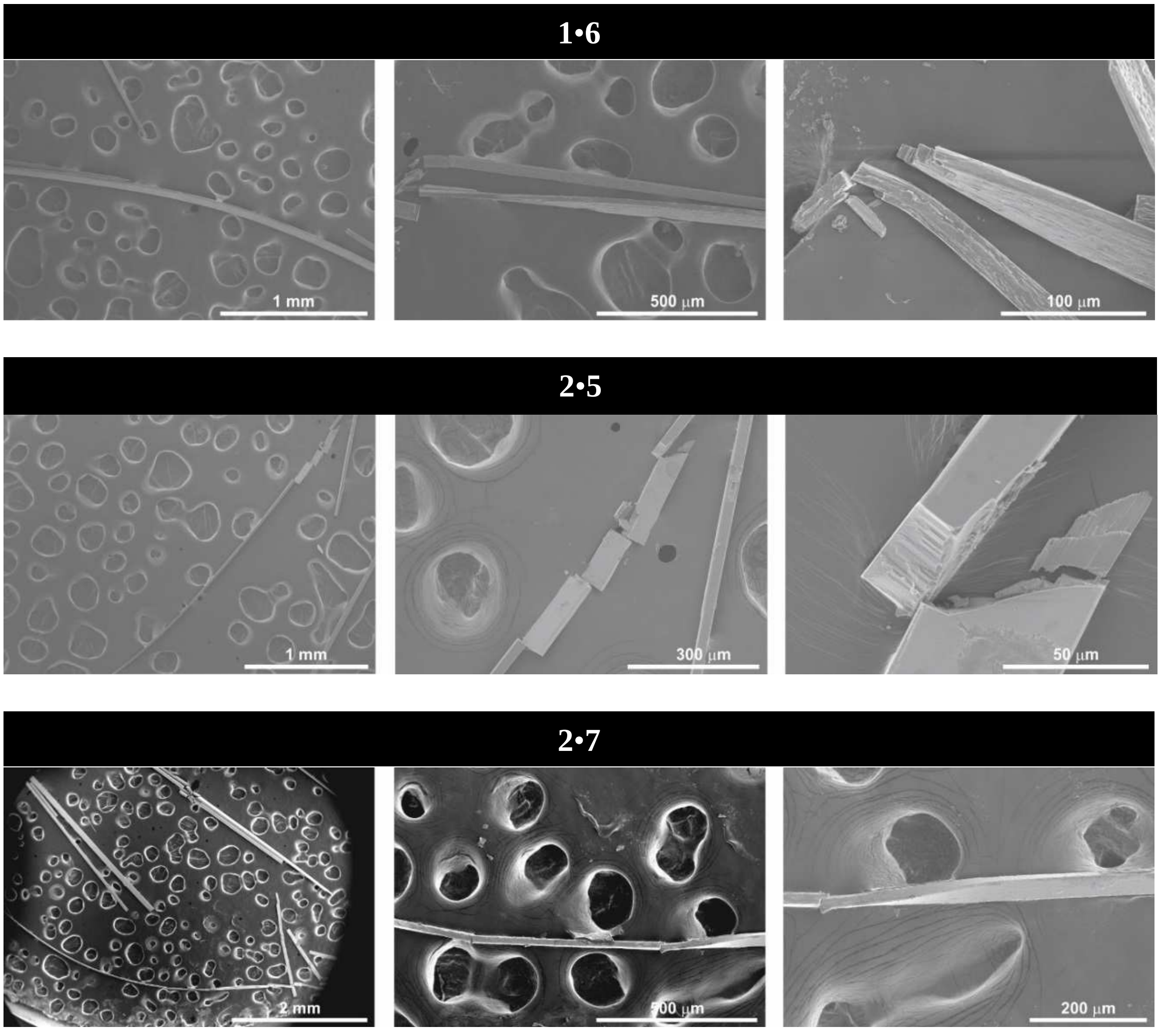

Supplementary Figure 14. SEM Images of mixed crystals. Bent elastic crystals were attached to a carbon tape used as a SEM substrate, and were intentionally fractured in order to expose the layers in the crystals.

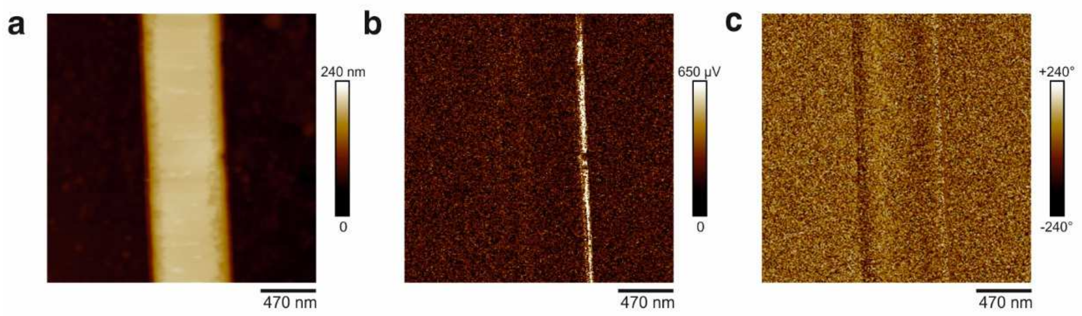

Supplementary Figure 15. Piezoresponse of 2 along the short axis. a, Topography image of another section of the same crystalline needle after rotating the sample 90 degrees. Lateral amplitude (**b**) and lateral phase (**c**) response of the short axis of the crystal.

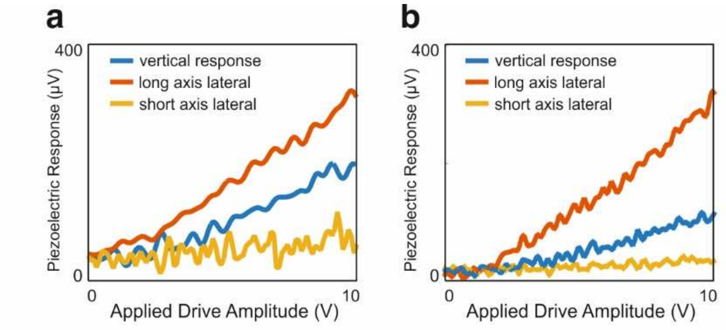

Supplementary Figure 16. Piezoresponse of 2 and 7 along three crystallographic directions. a and **b**: The voltage dependence of the piezoresponse of the various axes of the nanocrystal for **2** and **7** demonstrating the linear piezoelectric effect and dominance of the long axis piezoelectric effect.

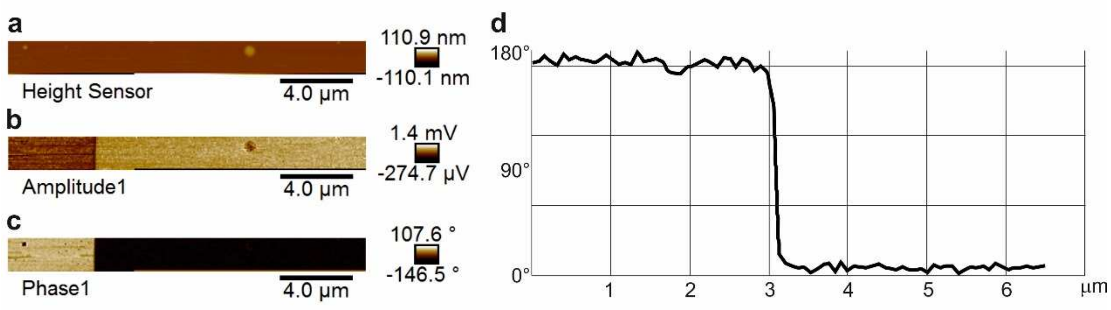

Supplementary Figure 17. PFM experiments preparation. PFM Image of a periodically poled lithium niobate (PPLN) crystal provided as a reference sample by Bruker Corp imaged at 35.0 kHz and 10Vpp. Height image of the surface (a), followed by the vertical amplitude (b), and the phase of the vertical signal (c). A section of the phase image is displayed, showing the 180 degree domain wall present in the poled sample (d).

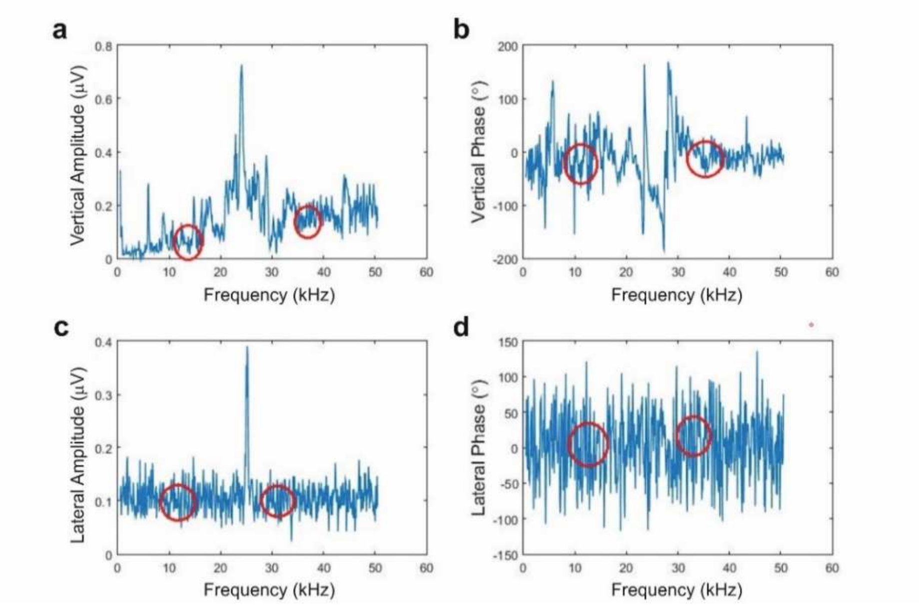

Supplementary Figure 18. PFM experiments preparation. Drive frequency sweeps of the SCM-PIT cantilever used in the experiments on a non-piezoelectric glass slide for the vertical amplitude (a), vertical phase (b), lateral amplitude (c), and lateral phase (d). As no easy reference sample exists for the calibration of lateral signals in PFM, these drive amplitudes serve as a good approximation for the background signal. Red circles represent areas of low background signal employed in this work.

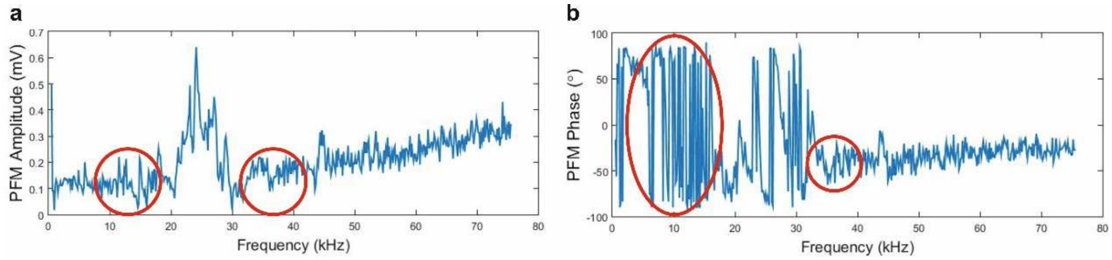

Supplementary Figure 19. PFM experiments preparation. Quantitative calculation of the background contribution for the cantilever employed performed from a PPLN sample. Both the vertical amplitude signal (a) and the vertical phase signal (b) have areas of low background contribution. Areas which are circled similarly in Supplementary Fig. 18 are circled here as well.

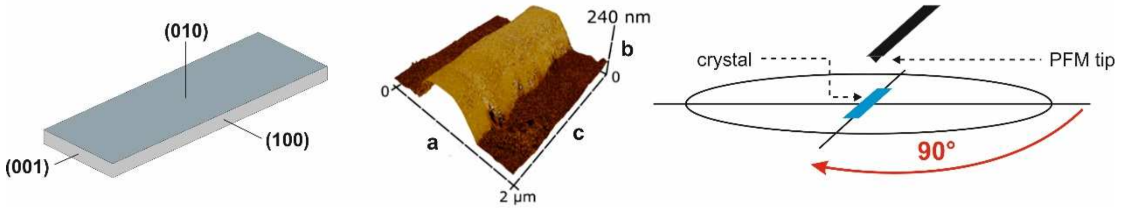

Supplementary Figure 20. PFM experiments on the crystals of 2. Crystal face indexing (left) of **2** and its three dimensional topographical image (middle) acquired using PFM, with the lateral phase signal as the colourmap. As the cantilever only has two degrees of freedom with which to probe the piezoelectric motion of the sample, physical alignment of either the long or short in-plane axis of the crystalline needle with the torsion of the cantilever was performed by rotating the sample 90 degrees to obtain the alternate axis (right).

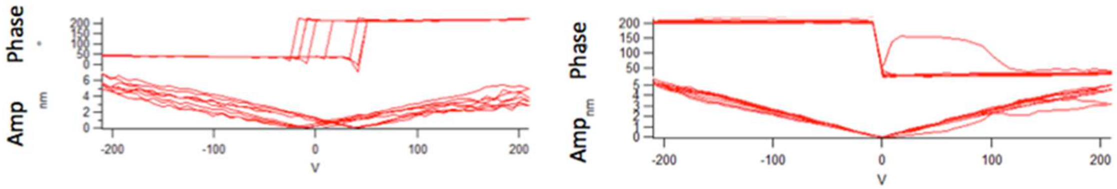

Supplementary Figure 21. Piezoelectric hysteresis loops of 2•7. Lateral amplitude and phase (left), and vertical amplitude and phase (right) hysteresis loops obtained by a continuous dc mode.

Supplementary Tables

Supplementary Table 1. Crystal data and structure refinement parameters for 1–6.

	**1**	**2**[1]	**3a**	**3b**	**4**[2]	**5**[2]	**6**
Empirical formula	$C_4H_4Cl_2N_2$	$C_4H_4Br_2N_2$	$C_4H_4I_2N_2$	$C_4H_4I_2N_2$	$C_3HCl_3N_2$	$C_3HN_2Br_3$	$C_3HBrCl_2N_2$
Formula weight / g mol^{-1}	150.99	239.90	333.89	333.89	171.41	304.79	215.87
Temperature / K	100	150	250	100	100	100	100
Wavelength / Å	0.71073	0.71073	0.71073	0.71073	1.54178	0.71073	0.71073
Crystal system	Orthorhombic	Orthorhombic	Orthorhombic	Orthorhombic	Orthorhombic	Orthorhombic	Orthorhombic
Space group	*Ama*2	*Ama*2	*Ama*2	*Ama*2	*Ama*2	*Ama*2	*Ama*2
a / Å	9.9314(8)	10.0598(11)	10.1538(3)	10.1862(2)	9.9548(15)	10.119(2)	9.9391(8)
b / Å	15.9458(12)	16.5717(19)	17.6311(6)	17.5316(3)	16.151(3)	16.618(3)	16.2112(15)
c / Å	3.7868(3)	4.0208(4)	4.4158(2)	4.3274(1)	3.7885(7)	3.9504(9)	3.8016(3)
V / $Å^3$	599.69(8)	670.30	790.53	772.79	609.11(18)	664.3(2)	612.53(9)
Z	4	4	4	4	4	4	4
D_{calc} / Mg m^{-3}	1.672	2.377	2.805	2.870	1.869	3.048	2.341
μ / mm^{-1}	0.96	11.99	7.87	8.05	12.70	18.11	7.46
F(000)	304	448	592	592	336	552	408
Crystal size / mm^3	0.35 × 0.07 × 0.04	0.58 × 0.06 × 0.04	0.48 × 0.11 × 0.01	0.37 × 0.25 × 0.13	0.47 × 0.06 × 0.01	0.85 × 0.12 × 0.03	0.15 × 0.05 × 0.02
θ range / °	3.3–29.6	5–27	3.1-35.6	4.0–36.5	7.1–63.6	4.0–32.6	2.5–30.1
Ranges of h, k, l	$-13 \leq h \leq 13$<br>$-22 \leq k \leq 22$<br>$-5 \leq l \leq 5$	$-12 \leq h \leq 13$<br>$-21 \leq k \leq 21$<br>$-5 \leq l \leq 5$	$-14 \leq h \leq 16$<br>$-28 \leq k \leq 28$<br>$-7 \leq l \leq 7$	$-16 \leq h \leq 17$<br>$-29 \leq k \leq 28$<br>$-7 \leq l \leq 6$	$-11 \leq h \leq 11$<br>$-18 \leq k \leq 17$<br>$-4 \leq l \leq 4$	$-15 \leq h \leq 15$<br>$-24 \leq k \leq 24$<br>$-5 \leq l \leq 5$	$-14 \leq h \leq 14$<br>$-20 \leq k \leq 22$<br>$-5 \leq l \leq 4$
Absorption correction	multi-scan	multi-scan	multi-scan	numerical	multi-scan	numerical	multi-scan
Reflections collected/unique	7476/887	4654/456	16859/1890	6531/1711	1150/434	5258/1138	3799/878
R_{int}	0.065	0.129	0.081	0.044	0.047	0.074	0.073
Refinement method	Full-matrix least-squares on F^2						
Data/restraints/parameters	887/1/41	456/3/41	1890/1/41	1711/1/42	434/1/40	1138/1/40	878/1/40
Goodness-of-fit on F^2	1.13	1.25	1.05	1.14	1.13	1.04	1.13
Final R indices [$I > 2\sigma(I)$]	R_1 = 0.0206<br>wR_2 = 0.0507	R_1 = 0.0511<br>wR_2 = 0.1253	R_1 = 0.0315<br>wR_2 = 0.0791	R_1 = 0.0261<br>wR_2 = 0.00724	R_1 = 0.0494<br>wR_2 = 0.1405	R_1 = 0.0363<br>wR_2 = 0.0853	R_1 = 0.0591<br>wR_2 = 0.1474
R indices (all data)	R_1 = 0.0218<br>wR_2 = 0.0513	R_1 = 0.0536<br>wR_2 = 0.1286	R_1 = 0.0412<br>wR_2 = 0.0846	R_1 = 0.0269<br>wR_2 = 0.0729	R_1 = 0.0505<br>wR_2 = 0.1413	R_1 = 0.0412<br>wR_2 = 0.0878	R_1 = 0.0639<br>wR_2 = 0.1512
$\Delta\rho_{max}/\Delta\rho_{min}$ / e $Å^{-3}$	0.28/-0.23	1.07/-1.16	0.75/-0.88	1.06/-1.46	0.87/-0.64	1.17/-1.55	1.48/-2.14
Flack parameter	0.08(5)	-	0.06(4)	0.11(7)	0.09(6)	-0.06(5)	0.02(3)
CCDC number	1425312	1030313	1425317	1425318	1425319	1425320	1425321

Supplementary Table 2. Crystal data and structure refinement parameters for 7–12.

	**7**	**8**	**9**	**10**	**11**[2]	**11 · MeOH**	**12**[1]
Empirical formula	$C_3HN_2ClBr_2$	$C_4H_4N_2Br_2$	$C_5H_6N_2Cl_2$	$C_5H_6N_2Br_2$	$C_3HI_3N_2$	$C_6H_2I_6N_4\cdot(CH_4O)$	$C_5H_7BrN_2$
Formula weight / g mol^{-1}	260.33	239.91	165.02	253.94	445.76	923.56	175.03
Temperature / K	100	100	100	100	100	250	150
Wavelength / Å	1.54178	0.71073	0.71073	0.71073	1.54178	1.54178	0.71073
Crystal system	Orthorhombic	Orthorhombic	Orthorhombic	Orthorhombic	Monoclinic	Monoclinic	Orthorhombic
Space group	*Ama*2	*Ama*2	*Pbca*	*Pbca*	$P2_1/c$	$P2_1/c$	*Pbcm*
a / Å	10.0186(7)	10.0665(5)	9.9749(7)	10.1725(4)	13.9023(10)	11.4563(3)	3.9495(2)
b / Å	16.4996(8)	16.6713(9)	8.6094(8)	8.7364(4)	22.1713(15)	9.1817(2)	15.8764(7)
c / Å	3.8923(2)	3.8891(2)	16.1480(13)	16.7335(7)	9.1820(7)	17.9844(5)	10.1544(4)
β / °	-	-	-	-	107.162(2)	94.6221(12)	-
V / Å^3	643.41(6)	652.68(6)	1386.8(2)	1487.12(11)	2704.2(3)	1885.60(8)	636.72(5)
Z	4	4	8	8	12	4	4
D_{calc} / Mg m^{-3}	2.687	2.442	1.581	2.268	3.285	3.253	1.826
μ / mm^{-1}	18.934	12.31	0.84	10.81	81.08	77.61	6.35
F(000)	480	448	672	960	2304	1608	344
Crystal size / mm^3	0.12 × 0.02 × 0.01	0.31 × 0.04 × 0.02	0.35 x 0.35 x 0.04	0.42 × 0.13 × 0.01	0.36 × 0.11 × 0.02	0.49 × 0.08 × 0.05	0.14 × 0.12 × 0.02
θ range / °	5.4–64.9	4.1–29.5	3.4–26.4	2.4–29.1	3.9–66.6	6.2–67.6	5–27
Ranges of h, k, l	$-10 \le h \le 11$<br>$-18 \le k \le 18$<br>$-4 \le l \le 4$	$-13 \le h \le 13$<br>$-22 \le k \le 22$<br>$-4 \le l \le 5$	$-12 \le h \le 12$<br>$-10 \le k \le 9$<br>$-20 \le l \le 20$	$-13 \le h \le 13$<br>$-11 \le k \le 13$<br>$-22 \le l \le 22$	$-13 \le h \le 16$<br>$-26 \le k \le 26$<br>$-10 \le l \le 10$	$-8 \le h \le 13$<br>$-10 \le k \le 10$<br>$-20 \le l \le 21$	$-5 \le h \le 5$<br>$-20 \le k \le 20$<br>$-12 \le l \le 13$
Absorption correction	multi-scan	multi-scan	multi-scan	multi-scan	multi-scan	integration	multi-scan
Reflections collected/unique	3880/584	9442/887	12960/1352	37909/1981	37209/4740	14684/3351	1300/745
R_{int}	0.048	0.030	0.037	0.062	0.075	0.054	0.020
Refinement method	Full-matrix least-squares on F^2						
Data/restraints/parameters	584/1/43	887/1/45	1352/0/83	1981/0/83	4740/0/211	3351/0/167	745/0/40
Goodness-of-fit on F^2	1.094	1.04	1.11	1.12	1.06	1.11	0.86
Final R indices [$I > 2\sigma(I)$]	R_1 = 0.0224<br>wR_2 = 0.0496	R_1 = 0.0143<br>wR_2 = 0.0311	R_1 = 0.0367<br>wR_2 = 0.0915	R_1 = 0.0316<br>wR_2 = 0.0683	R_1 = 0.0726<br>wR_2 = 0.1961	R_1 = 0.0641<br>wR_2 = 0.1622	R_1 = 0.0382<br>wR_2 = 0.0985
R indices (all data)	R_1 = 0.0238<br>wR_2 = 0.0500	R_1 = 0.0155<br>wR_2 = 0.0314	R_1 = 0.0412<br>wR_2 = 0.0955	R_1 = 0.0475<br>wR_2 = 0.0760	R_1 = 0.0763<br>wR_2 = 0.2052	R_1 = 0.0654<br>wR_2 = 0.1641	R_1 = 0.0518<br>wR_2 = 0.1046
$\Delta\rho_{max}/\Delta\rho_{min}$ / e Å^{-3}	0.31/-0.49	0.51/-0.28	0.41/-0.28	0.62/-0.78	3.21/-2.52	1.86/-3.12	0.48/-0.50
Flack parameter	-0.05(6)	0.02(2)	-	-	-	-	-
CCDC number	1425322	1425323	1425324	1425325	1425326	1425327	960285

Supplementary Table 3. Bond critical points of haloimidazoles. The characteristic of the bond critical point (BCP) of the intermolecular halogen-halogen contacts of selected haloimidazoles. Symbols: ρ(r), the electron density; $\nabla^2\rho$(r), the Laplacian of the electron density; G(r), the electron kinetic energy density [a.u.]; V(r), the electron potential energy density [a.u.]; $DE^G = 0.429G$(r), the dissociation energy [kcal/mol]; ε^{AIM}, the ellipticity; d, the deviation from linearity [Å].

Compound	Atoms	ρ(r)	$\nabla^2\rho$(r)	G(r)	V(r)	DE^G	ε^{AIM}	d	Bond?
**1**	Cl2···Cl3	0.0069	0.0282	0.0054	-0.0038	0.00232	0.1152	0.0205	√
**1**	Cl1···Cl4	0.0069	0.0279	0.0054	-0.0038	0.00232	0.1184	0.0212	√
**2**	Br2···Br3	0.0079	0.0267	0.0053	-0.0039	0.00227	0.1180	0.0138	√
**2**	Br1···Br4	0.0079	0.0270	0.0054	-0.0040	0.00232	0.1155	0.0129	√
**4**	Cl2···Cl3	0.0065	0.0268	0.0051	-0.0035	0.00219	0.1167	0.0208	√
**4**	Cl1···Cl4	0.0066	0.0269	0.0051	-0.0036	0.00219	0.1176	0.0220	√
**4**	Cl1···Cl5	0.0038	0.0137	0.0025	-0.0016	0.00107	0.1325	0.0240	×
**4**	Cl2···Cl5	0.0038	0.0137	0.0025	-0.0016	0.00107	0.1354	0.0251	×
**4**	Cl3···Cl5	0.0036	0.0114	0.0021	-0.0014	0.00090	0.0590	0.0021	×
**4**	Cl4···Cl5	0.0036	0.0114	0.0021	-0.0014	0.00090	0.0525	0.0016	×
**4**	Cl7···Cl8	0.0036	0.0113	0.0021	-0.0013	0.00090	0.0397	0.0016	×
**4**	Cl6···Cl8	0.0037	0.0113	0.0021	-0.0013	0.00090	0.0480	0.0020	×

Supplementary Table 4. Halogen-halogen interactions analysis. Geometric parameters, θ_1 and θ_1 angles, of halogen-halogen interactions of **1–8** haloimidazoles and their classification.[a] Positional disorder at 4 and 5 positions.

Compound	X···X	θ_1 / °	θ_2 / °	$\|\theta_1 - \theta_2\|$	X···X interaction type
**1**	Cl···Cl	162.13	127.29	34.84	Type II (halogen bond)
**2**	Br···Br	162.51	122.75	39.76	Type II (halogen bond)
**3**	I···I	159.44	120.16	39.28	Type II (halogen bond)
**4**	Cl···Cl	162.24	126.44	35.80	Type II (halogen bond)
**5**	Br···Br	161.55	125.36	36.19	Type II (halogen bond)
**6**	Cl···Cl	162.12	127.53	34.59	Type II (halogen bond)
**7**[a]	Br···Br	160.52	125.61	34.91	Type II (halogen bond)
	Cl···Cl	167.97	126.83	41.14	Type II (halogen bond)
	Br···Cl	166.28	127.72	38.56	Type II (halogen bond)
**8**	Br···Br	163.02	128.57	34.45	Type II (halogen bond)

Supplementary Table 5. Bond critical points of hexachlorobenzene. The characteristic of BCP of the intermolecular contacts of hexachlorobenzene. See Supplementary Table 3 for symbol explanation.

Atoms	ρ(r)	$\nabla^2\rho$(r)	G(r)	V(r)	DE^G	ε^{AIM}	d	Stability?
Cl12···Cl25	0.0034	0.0111	0.0021	-0.0014	0.00090	0.3920	0.0202	×
Cl7···Cl13	0.0059	0.0245	0.0046	-0.0032	0.00197	0.0556	0.0163	√
Cl24···Cl25	0.0063	0.0261	0.0050	-0.0034	0.00215	0.0977	0.0094	√
Cl7···Cl24	0.0054	0.0199	0.0037	-0.0025	0.00159	0.1068	0.0161	×
Cl23···Cl25	0.0038	0.0133	0.0025	-0.0016	0.00107	0.1510	0.0162	×
Cl12···Cl32	0.0057	0.0195	0.0037	-0.0025	0.00159	0.2021	0.0008	×
Cl11···Cl32	0.0034	0.0111	0.0021	-0.0014	0.00090	0.3939	0.0197	×

Supplementary Table 6. Parameters of the Cole-Cole relation for selected temperatures for 1 and 3.

**1**					**3**				
T (K)	$\tau \times 10^7$ (s)	ε_0	ε_∞	α	T (K)	$\tau \times 10^7$ (s)	ε_0	ε_∞	α
270	4.80	1.623	1.180	0.697	140	37.0	3.142	2.782	0.665
275	3.99	1.630	1.190	0.674	155	13.0	3.153	2.782	0.626
280	3.15	1.626	1.199	0.646	170	6.29	3.164	2.794	0.577
285	2.59	1.628	1.198	0.635	185	3.23	3.157	2.810	0.505
290	2.19	1.624	1.201	0.622	200	2.00	3.160	2.819	0.444

Supplementary Table 7. **Parameters of the Havriliak-Negami relation for selected temperatures for 2.**

**2**						**2**					
T (K)	$\tau \times 10^4$ (s)	ε_0	ε_∞	α	β	T (K)	$\tau \times 10^4$ (s)	ε_0	ε_∞	α	β
265	28.0	3.166	1.237	0.902	0.620	272	2.60	3.450	1.149	0.570	0.789
267	18.3	3.257	1.241	0.852	0.629	273	1.89	3.435	1.146	0.608	0.595
269	12.0	3.378	1.224	0.843	0.556	274	1.16	3.428	1.155	0.567	0.606
270	8.34	3.316	1.229	0.792	0.587	275	0.83	3.442	1.097	0.526	0.598
271	6.00	3.450	1.182	0.712	0.578	276	0.69	3.432	1.134	0.535	0.596

Supplementary Table 8. Mixed crystals preparation. Amounts of materials taken to prepare haloimidazoles solid solutions. Symbols: m^1 and n^1 – mass and moles of component 1; m^2 and n^2 – mass and moles of component 2.

**Solution**	**Component 1 (disturber)**	**Component 2 (basic structure)**	**Mass / mg**	**Moles / mmol**
**1•4**	**1**	**4**	m^1 = 7.0<br>m^2 = 7.9	n^1 = 0.046<br>n^2 = 0.046
**1•5**	**1**	**5**	m^1 = 14.6<br>m^2 = 14.0	n^1 = 0.097<br>n^2 = 0.046
**1•6**	**1**	**6**	m^1 = 16.4<br>m^2 = 10.2	n^1 = 0.109<br>n^2 = 0.047
**1•7**	**1**	**7**	m^1 = 12.2<br>m^2 = 10.5	n^1 = 0.081<br>n^2 = 0.040
**2•4**	**2**	**4**	m^1 = 10.2<br>m^2 = 7.10	n^1 = 0.043<br>n^2 = 0.041
**2•5**	**2**	**5**	m^1 = 10.4<br>m^2 = 12.9	n^1 = 0.043<br>n^2 = 0.042
**2•6**	**2**	**6**	m^1 = 10.5<br>m^2 = 4.70	n^1 = 0.044<br>n^2 = 0.022
**2•7**	**2**	**7**	m^1 = 10.1<br>m^2 = 10.6	n^1 = 0.042<br>n^2 = 0.041

Supplementary Table 9. **Crystal data and structure refinement parameters for 1•4, 1•5, 1•6, 1•7, 2•4, 2•5, 2•6, and 2•7 solid solutions**.

	**1•4**	**1•5**	**1•6**	**1•7**	**2•4**	**2•5**	**2•6**	**2•7**
Empirical formula	$C_{3.39}H_{2.16}Cl_{2.61}N_2$	$C_{3.65}H_{2.97}N_2Cl_{1.35}Br$	$C_{3.7}H_{3.1}N_2Cl_2Br_{0.3}$	$C_{3.7}H_{3.11}N_2Cl_{1.73}Br_{0.56}$	$C_{3.37}H_{2.11}N_2Cl_{1.48}Br_{1.15}$	$C_{3.51}H_{2.52}N_2Br_{2.49}$	$C_{3.69}H_{3.08}N_2Cl_{0.73}Br_{1.58}$	$C_{3.55}H_{2.66}N_2Cl_{0.58}Br_{1.87}$
Formula weight / g mol^{-1}	163.50	200.55	170.62	181.88	214.78	271.87	227.30	243.27
Temperature / K	100	100	100	100	100	100	100	100
Wavelength / Å	0.71073	0.71073	0.71073	1.54178	0.71073	0.71073	1.54178	0.71073
Crystal system	Orthorhombic	Orthorhombic	Orthorhombic	Orthorhombic	Orthorhombic	Orthorhombic	Orthorhombic	Orthorhombic
Space group	*Ama*2	*Ama*2	*Ama*2	*Ama*2	*Ama*2	*Ama*2	*Ama*2	*Ama*2
a / Å	9.9471(8)	9.971(2)	9.9274(4)	9.9503(6)	9.9970(8)	10.033(4)	10.0268(6)	10.0275(5)
b / Å	16.0705(11)	16.255(4)	16.0510(7)	16.2127(12)	16.3862(14)	16.524(7)	16.4133(10)	16.4911(8)
c / Å	3.7773(3)	3.8480(8)	3.7886(2)	3.8233(3)	3.8772(4)	3.9323(15)	3.9226(2)	3.9218(2)
V / $Å^3$	603.82(8)	623.7(2)	603.69(5)	616.78(8)	635.14(10)	651.9(4)	645.55(6)	648.53(6)
Z	4	4	4	4	4	4	4	4
D_{calc} / Mg m^{-3}	1.799	2.152	1.877	1.959	2.246	2.770	2.339	2.492
μ (mm^{-1})	1.226	7.006	2.958	11.748	7.909	15.343	14.769	11.826
F(000)	324	386	335	354	407	499	427.0	453
Crystal size / mm^3	0.70 × 0.08 × 0.04	0.50 × 0.04 × 0.04	0.41 × 0.11 × 0.08	0.10 × 0.03 × 0.02	0.51 × 0.04 × 0.03	0.60 × 0.08 × 0.04	0.60 × 0.05 × 0.02	0.47 × 0.07 × 0.02
θ range / º	3.3–30.5	2.5–31.0	4.8–35.0	5.5–66.2	4.8–30.6	2.5–26.4	5.4–64.6	4.8–35.4
Ranges of *h*, *k*, *l*	$-14 \le h \le 14$<br>$-22 \le k \le 22$<br>$-5 \le l \le 5$	$-14 \le h \le 14$<br>$-23 \le k \le 23$<br>$-5 \le l \le 5$	$-16 \le h \le 16$<br>$-25 \le k \le 25$<br>$-5 \le l \le 6$	$-11 \le h \le 11$<br>$-16 \le k \le 18$<br>$-4 \le l \le 4$	$-14 \le h \le 14$<br>$-23 \le k \le 22$<br>$-5 \le l \le 5$	$-11 \le h \le 12$<br>$-20 \le k \le 20$<br>$-4 \le l \le 2$	$-11 \le h \le 11$<br>$-17 \le k \le 18$<br>$-4 \le l \le 4$	$-16 \le h \le 16$<br>$-26 \le k \le 26$<br>$-6 \le l \le 6$
Absorption correction	multi-scan	multi-scan	numerical	multi-scan	numerical	multi-scan	multi-scan	multi-scan
Refl. collected/unique	3837/937	5385/1028	5787/1283	2370/545	5077/963	2361/517	3988/551	12492/1540
R_{int}	0.01011	0.052	0.053	0.028	0.079	0.026	0.027	0.032
Refinement method	Full-matrix least-squares on F^2							
Data/restraints/parameters	937/1/44	1028/1/52	1283/1/45	545/1/48	963/1/48	517/1/45	551/1/49	1540/1/49
Goodness-of-fit on F^2	1.06	1.06	1.09	1.07	0.982	1.09	1.08	1.05
Final R indices [$I > 2\sigma(I)$]	R_1 = 0.0405<br>wR_2 = 0.0941	R_1 = 0.0284<br>wR_2 = 0.0625	R_1 = 0.0300<br>wR_2 = 0.0683	R_1 = 0.0234<br>wR_2 = 0.0583	R_1 = 0.0321<br>wR_2 = 0.0690	R_1 = 0.0197<br>wR_2 = 0.0475	R_1 = 0.0139<br>wR_2 = 0.0328	R_1 = 0.0192<br>wR_2 = 0.0349
R indices (all data)	R_1 = 0.0474<br>wR_2 = 0.0986	R_1 = 0.0352<br>wR_2 = 0.0648	R_1 = 0.0354<br>wR_2 = 0.0694	R_1 = 0.0248<br>wR_2 = 0.0589	R_1 = 0.0415<br>wR_2 = 0.0715	R_1 = 0.0203<br>wR_2 = 0.0478	R_1 = 0.0140<br>wR_2 = 0.0329	R_1 = 0.0253<br>wR_2 = 0.0360
$\Delta\rho_{max}/\Delta\rho_{min}$ / e $Å^{-3}$	0.34/-0.41	0.43/-0.50	0.40/-0.29	0.36/-0.23	0.75/-0.83	0.50/-0.76	0.20/-0.25	0.49/-0.40
Flack parameter	-0.7(2)	0.023(19)	0.04(2)	-0.05(4)	0.03(3)	0.07(4)	0.09(5)	0.012(19)
CCDC number	1425308	1425309	1425310	1425322	1425313	1425314	1425315	1425316

Supplementary Table 10. Calculated (HPLC) and experimental (X-ray) components ratio of 1•5 crystals.[a]

1•5 mixed in 2:1 ratio														
	Crystal 1		Crystal 2		Crystal 3		Crystal 4		Crystal 5		average		X-ray	
Compound	1	5	1	5	1	5	1	5	1	5	1	5	1	5
Peak area [arb.u.]	389.2	280.2	197.1	137.3	509.7	376.5	396.5	284.3	322.3	226.4	—	—	—	—
$c \times 10^3$ [mol dm^{-3}]	0.138	0.076	0.072	0.041	0.179	0.100	0.140	0.077	0.115	0.063	—	—	—	—
Ratio [%]	64	36	64	36	64	36	64	36	65	35	64±1	36±1	66	34

[a] Calculated ratio is compared with the ratio for C5/Br5 atoms in the crystal structure (*cf.* Supplementary Figure 10).

Supplementary Table 11. Calculated (HPLC) and experimental (X-ray) components ratio of 1•6 crystals.

1•6 mixed in 2:1 ratio														
	Crystal 1		Crystal 2		Crystal 3		Crystal 4		Crystal 5		average		X-ray	
Compound	1	6	1	6	1	6	1	6	1	6	1	6	1	6
Peak area [arb.u.]	824.2	474.7	308.4	135.5	585.9	351.8	590.4	356.5	1053.4	631.9	—	—	—	—
$c \times 10^3$ [mol dm^{-3}]	0.286	0.140	0.110	0.045	0.205	0.106	0.206	0.107	0.364	0.184	—	—	—	—
Ratio [%]	67	33	71	29	66	34	66	34	66	34	67±1	33±1	70	30

Supplementary Table 12. Calculated (HPLC) and experimental (X-ray) components ratio of 1•7 crystals.[a]

1•7 mixed in 2:1 ratio														
	Crystal 1		Crystal 2		Crystal 3		Crystal 4		Crystal 5		average		X-ray	
Compound	1	7	1	7	1	7	1	7	1	7	1	7	1	7
Peak area [arb.u.]	1320.3	836.3	761.7	462.2	460.2	307.1	536.2	349.9	513.2	333.1	—	—	—	—
$c \times 10^3$ [mol dm^{-3}]	0.455	0.199	0.264	0.115	0.162	0.080	0.188	0.089	0.180	0.085	—	—	—	—
Ratio [%]	70	30	70	30	67	33	68	32	68	32	69±1	31±1	71	29

[a] Calculated ratio is compared with the ratio for C5/Br5 atoms in the crystal structure (*cf.* Supplementary Figure 10).

Supplementary Table 13. Calculated (HPLC) and experimental (X-ray) components ratio of 2•5 crystals.

2•5 mixed in 1:1 ratio														
	Crystal 1		Crystal 2		Crystal 3		Crystal 4		Crystal 5		average		X-ray	
Compound	2	5	2	5	2	5	2	5	2	5	2	5	2	5
Peak area [arb.u.]	457.8	534.4	253.2	290.7	918.8	1089.6	533.6	583.8	533.8	628.8	—	—	—	—
$c \times 10^3$ [mol dm^{-3}]	0.150	0.140	0.083	0.079	0.303	0.278	0.170	0.152	0.175	0.163	—	—	—	—
Ratio [%]	52	48	51	49	52	48	53	47	52	48	52±1	48±1	51	49

Supplementary Table 14. Calculated (HPLC) and experimental (X-ray) components ratio of 2•6 crystals.[a]

2•6 mixed in 2:1 ratio														
	Crystal 1		Crystal 2		Crystal 3		Crystal 4		Crystal 5		average		X-ray	
Compound	2	6	2	6	2	6	2	6	2	6	2	6	2	6
Peak area [arb.u.]	495.3	239.5	624.3	355.1	248.0	131.2	370.2	172.7	375.9	182.8	—	—	—	—
$c \times 10^3$ [mol dm^{-3}]	0.163	0.074	0.205	0.107	0.081	0.044	0.121	0.056	0.123	0.058	—	—	—	—
Ratio [%]	69	31	66	34	65	35	69	31	68	32	67±1	33±1	69	31

[a] Calculated ratio is compared with the ratio for C5/Br5 atoms in the crystal structure (*cf.* Supplementary Figure 10).

Supplementary Table 15. Calculated (HPLC) and experimental (X-ray) components ratio of 2•7 crystals.[a]

2•7 mixed in 1:1 ratio														
	Crystal 1		Crystal 2		Crystal 3		Crystal 4		Crystal 5		average		X-ray	
Compound	2	7	2	7	2	7	2	7	2	7	2	7	2	7
Peak area [arb.u.]	959.0	984.4	539.2	604.7	294.7	315.8	691.9	746.0	803.1	895.4	—	—	—	—
$c \times 10^3$ [mol dm^{-3}]	0.316	0.233	0.177	0.147	0.096	0.082	0.228	0.179	0.265	0.213	—	—	—	—
Ratio [%]	58	42	55	45	54	46	56	44	55	45	56±1	44±1	55	45

[a] Calculated ratio is compared with the ratio for C5/Br5 atoms in the crystal structure (*cf.* Supplementary Figure 10).

Supplementary Table 16. Nanoindentation tests. Elastic modulus (*E*) and hardness (*H*) of **1•4**, **1•5**, **1•6**, **1•7**, **2•4**, **2•5**, **2•6**, and **2•7** solid solution crystals.

Solid solution	Parameters	Test 1	Test 2	Test 3	Test 4	Test 5	average
**1•4**	*E* [GPa]	0.27	0.39	0.32	0.32	0.25	0.31±0.02
	H [MPa]	76.3	66.6	84.9	90.8	71.2	77.9±4.4
**1•5**	*E* [GPa]	0.38	0.20	0.37	0.21	0.20	0.27±0.04
	H [MPa]	89.0	60.1	82.9	51.3	53.3	67.3±7.8
**1•6**	*E* [GPa]	0.26	0.18	0.17	0.17	0.15	0.19±0.02
	H [MPa]	69.7	63.9	60.2	57.4	54.4	61.1±2.7
**1•7**	*E* [GPa]	0.29	0.29	0.27	0.24	0.22	0.26±0.01
	H [MPa]	63.5	52.9	66.7	62.1	76.4	64.3±3.8
**2•4**	*E* [GPa]	0.10	0.11	0.09	0.09	0.09	0.10±0.05
	H [MPa]	29.2	34.7	29.7	75.3	24.9	38.7±9.2
**2•5**	*E* [GPa]	0.17	0.27	0.22	0.16	0.16	0.20±0.05
	H [MPa]	55.0	68.8	53.2	51.8	56.9	57.1±3.0
**2•6**	*E* [GPa]	0.24	0.19	0.20	0.23	0.24	0.22±0.01
	H [MPa]	73.5	48.3	54.9	46.7	59.0	56.5±4.8
**2•7**	*E* [GPa]	0.17	0.29	0.21	0.16	0.21	0.21±0.02
	H [MPa]	46.1	59.8	53.9	60.7	52.5	54.6±2.7

Supplementary Table 17. **PFM experiments preparation.** Comparison of calculated background vectors from both the glass slide and the PPLN samples. Since vertical amplitudes compare well between the methods for 35 kHz, these values of background amplitude were utilized in the correction of vertical and lateral images in the main section.

f (kHz)	PPLN Vert. Amp.	PPLN Vert. Phase	Glass Vert. Amp.	Glass Vert. Phase	Glass Lat. Amp.	Glass Lat. Phase
**12.5**	0.1606 mV	-81.2538°	0.0846 mV	-24.5028°	0.1343 mV	-7.8750°
**35.0**	0.1678 mV	-56.8581°	0.1604 mV	-30.0850°	0.1160 mV	56.6928°

Supplementary Notes

Supplementary Note 1. Theoretical calculations

The values of the electron density, $\rho(\mathrm{r})$, and the Laplacian of the electron density, $\nabla^2\rho(\mathrm{r})$, at the BCP of halogen-halogen interactions (Supplementary Table 3) between molecules being related by a 2-fold screw axis (m_1 and m_4, and m_4 and m_3 in Fig. 4 and Supplementary Fig. 6) are in accordance with the values for other weak intermolecular contacts, e.g., hydrogen and dihydrogen bonds (criteria range: 0.002–0.035 a.u. and 0.024–0.139 a.u. for $\rho(\mathrm{r})$ and $\nabla^2\rho(\mathrm{r})$,

respectively)[3-5]. Higher values of $\rho(\mathrm{r})$ for **2** in comparison to **1** indicate that the substitution of chlorine with bromine increases substantially the strength of the halogen-halogen interactions. The introduction of a halogen atom instead of the methyl group in the second position of imidazole does not provide better stabilisation of the structure. Since contacts involving Cl5 and Cl8 atoms are described by approximately two times lower values of $\rho(\mathrm{r})$ and $\nabla^2\rho(\mathrm{r})$, they are not considered chemical bonds. Low values of the dissociation energy[6] of these contacts, DE^G, confirm the lack of interaction.

Information about stability of the X···X interactions, the presence of which were confirmed by the AIM method, can be found in other parameters, such as ellipticity, $\varepsilon^{\mathrm{AIM}}$, and deviation from linearity[7], *d*. Both of them exceed the values that characterize stable intermolecular interactions[4,8]. This fact indicates substantial susceptibility of these bonds to rupture in case of any structural changes. The process of breaking these bonds and regaining their original position after being deformed is believed to make an important contribution towards the mixed crystals of haloimidazoles acquiring elastic properties. In order to prove this hypothesis, AIM analysis of a reference crystal structure in which halogen-halogen interactions are definitely broken/restored on account of strong structural changes was performed. Hexachlorobenzene, whose crystals can be deformed plastically by applying external force, owes its plastic properties to the restorative effect of the Cl···Cl interactions which act cohesively to recover the adhesion between the hexachlorobenzene layers. The BCP parameters of Cl···Cl contacts in a hexachlorobenzene structural motif containing three molecules (Fig. 4b) are collected in Supplementary Table 5. Out of seven bond paths that were identified by the AIM method, two were found to be the interactions that stabilize the structure, and five, because of low values $\nabla^2\rho(\mathrm{r})$ and high values of $\varepsilon^{\mathrm{AIM}}$, can be easily ruptured. This fact not only explains the ease in which the layers of π-stacked molecules can glide on top of each other to alleviate the strain, but also gives an important clue as to why mixed crystals of haloimidazoles show elastic properties instead of plastic ones.

Supplementary Note 2. Analysis of halogen···halogen interactions

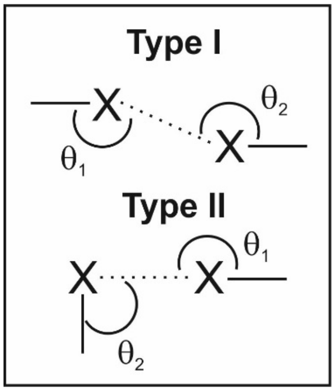

Two varieties of halogen-halogen contacts are known — Type I and Type II. While Type I interactions are symmetrical, van der Waals in nature, and are attributed to close packing, Type II interactions arise form electrostatic attractions between polarized halogen atoms. Recently, the following geometric criteria for classification of Type I and Type II contacts were suggested — contacts with $0° \leq |\theta_1 - \theta_2| \leq 15°$ are Type I; contacts with $30° \leq |\theta_1 - \theta_2|$ are Type II; contacts with $15° \leq |\theta_1 - \theta_2| \leq 30°$ are quasi-Type I/Type II. Since Type II halogen-halogen contacts involve interactions between the electrophilic region of one halogen atom with the nucleophilic region of the other, these contacts qualify as being true halogen bonds according to the current IUPAC definition. An analysis (Supplementary Table 4) of the halogen-halogen interactions of eight isostructural haloimidazoles, **1–8**, has shown that Type II halogen-halogen interactions (halogen bonds) are present in the crystal structures of haloimidazoles.

Supplementary Note 3. Electric permittivity measurements

*Origin of the relaxation processes***.** Electric permittivity is related directly to the polarisation of the system, and therefore to the dipole moment of molecules in a crystal. In the case of the hydrogen-bonded haloimidazoles, two possible mechanisms that may change the value of the dipole moment in either *x*, *y* or *z* crystallographic direction need to be considered: proton transfer in N–H···N hydrogen bridges and the tilting of molecules from their original positions when an alternating electric field is applied. While the proton motion is fast and occurs usually in a high frequency region, the libration motion of molecules seem to be a more likely mechanism. The analysis of the closest environment of the molecule in the crystal lattice (Fig. 5) revealed that each molecule is surrounded by eight halogen atoms that form a cube inside which the molecule is located. While halogen and N–H···N bonds stabilize the molecule on one side of the cube, no interactions are present on the other side of the cube, a situation which is reflected by the significantly larger thermal ellipsoid of the carbon atom of the CH_3 group. Therefore, the molecular mobility in the crystal lattice is not completely restricted, allowing the CH_3 group to perform a precessive motion or the whole molecule to exhibit a

swinging motion, which manifests itself as the relaxation processes when an alternating electric field is applied.

Supplementary Note 4. Piezoresponse force microscopy

Piezoresponse Force Microscopy (PFM) is a variation of contact Atomic Force Microscopy (AFM) in which a conductive nanoscale stylus is scanned across the surface. At each pixel an AC electric field is applied between the sample and tip, and the corresponding mechanical excitation is detected through the vibration of the cantilever. For a piezoelectric sample, the oscillating electric field induces a linear mechanical strain, which is related through the third order piezoelectric tensor for the material. Thus, we can image the piezoelectric response at a fixed voltage over the sample and image the linear response of the material quantitatively by sweeping the voltage at fixed points, all while correlating with the topography.

The mechanical deflection of the cantilever in response to the electromechanical coupled strain of the material is detected through the use of a lock-in amplifier. The original AC driving signal is used as a reference signal in two lock-in amplifiers, which detect both the vertical deflection of the cantilever and the lateral in-plane torsion. Since the piezoelectric response is a third order tensor, we can have an in-plane response from a vertical field that is forbidden from both the second order electrostrictive response and the electrostatic response. The magnitude and the phase of the mechanical oscillation are recorded point-by-point, and together yield quantitative information about the orientation of the piezoelectric response of the material.

In order to ensure the instrument is operating properly, we first of all image a periodically poled lithium niobate (PPLN) crystal in which ferroelectric domains of opposite orientation have been written electrically into the sample. Between these domains the orientation of the net polarisation flips 180 degrees out of the plane, resulting in a corresponding 180 degree phase shift of the vertical signal. This phase shift is displayed in Supplementary Fig. 17 where parallel stripe 180 degree domain structures are easily visualized with the same cantilever imaged directly before performing the experiments in this work.

As we are often detecting picometer scale vibrations in PFM, the technique is very sensitive to external vibrations in the surrounding environment, a situation which can often dominate the signal. Thus, it is of key importance to, first of all, choose an AC excitation frequency with a low background contribution, and secondly, furthermore, to subtract the remaining signal from images. Following the procedure of Jungk et al.[9], we first of all performed frequency sweeps

of both the vertical and lateral phase and amplitude signals on a grounded glass slide. Five sweeps were captured and averaged for each channel. As glass is both non-piezoelectric and non-ferroelectric, the recorded frequency spectrum should be a pure measurement of the background signal. In reality, although the phase component often oscillates widely in such samples, it still remains an effective way of choosing an operating frequency with limited background contribution and the only simple way of measuring the background contribution of the lateral signal. In the next section, the vertical signal for the frequencies highlighted (Supplementary Fig. 18) is compared to the background spectrum from a quantitative calibration method in order to justify the use of the lateral background amplitude when correcting images.

Next, we measured the frequency dependent quadrature and in-phase signals for a +*c* and –*c* domain on the previously imaged PPLN crystal. At the centre of the positively and negatively poled regions, sweeps for amplitude and phase were collected and averaged over five samples. When operating far from contact resonance[9]—typically in the hundreds of kHz range—the majority of the peaks in the spectrum, when combined appropriately, arise from spurious background vibrations. All data collected in this research and reported in this article were obtained in the low frequency regime far from the mechanical resonance in order to avoid issues with topographical cross-talk. Thus, the signals from the opposite domains are added and averaged in order to obtain the pure background signal for the vertical signal of the system, which is displayed in Supplementary Fig. 19.

Finally, the magnitude and phase of the background signal were compared (Supplementary Table 17) between the PPLN sample and the glass slide. Good agreement is found between the measurements for 35.0 kHz. Therefore, images presented in this article were corrected using the background amplitude for the vertical and lateral amplitude images recorded at the same deflection setpoint and $10V_{pp}$. As the phase signal is highly variable for a non-piezoelectric sample and no easy calibration sample exists for the lateral signal, the phase images are presented untouched.

The geometry employed in the experiment was prepared such that the AFM probe engaged vertically on the shortest axis of the crystal, corresponding to the crystal face (010) or (100) for **2** and **7,** respectively. From the solid-state structure, obtained by X-ray crystallography, we can determine the orientation of the crystal from the ratio of the physical dimensions of each crystal

(Supplementary Fig. 20). The deflection sensitivity of the cantilever was measured by ramping the cantilever against a hard silicon dioxide substrate and measuring the slope of the linear deflection segment. The deflection sensitivity was recorded to be 106 nm V^{-1} which compares well with the nominal value of 110 nm V^{-1} for SCM PIT provided by Bruker. This value is used in the main section of the article to convert mV V^{-1} piezoelectric coefficients to a value in the traditional units of nm V^{-1}.

Supplementary Methods

Materials & General Methods & Instrumentation

4,5-Dichloro-2-methylimidazole (**1**) (Alfa Aesar, 97%) and 4,5-dibromo-2-methyl-imidazole (**2**) (Combi-Blocks, 98%) were used as received. 2,4,5-Trichloroimidazole (**4**)[10], 2,4,5-tribromoimidazole (**5**)[11], 2,4-dibromo-5-methylimidazole (**8**)[12], 4,5-dibromo-2-ethyl-imidazole (**10**)[13], 2,4,5-triiodoimidazole (**11**)[14], and 2-bromo-4,5-dimethyl-imidazole (**12**)[15] were prepared according to literature procedures[10-15]. All starting materials and reagents were purchased from commercial suppliers and used without further purification. Nuclear magnetic resonance (NMR) spectra were recorded at 298 K on Bruker Avance 500 spectrometer, with working frequencies of 500 MHz for ^{1}H, and 125 MHz ^{13}C nuclei. Chemical shifts are reported in ppm relative to the signals corresponding to the residual non-deuterated solvent. All ^{13}C NMR spectra were recorded with the simultaneous decoupling of proton nuclei. Electrospray ionisation (ESI) mass spectra were obtained on an Agilent 6210 LCTOF high-resolution mass spectrometer. The phase situation of haloimidazoles was investigated using a Perkin Elmer 8500 Differential Scanning Calorimeter (DSC) calibrated with *n*-heptane and indium. Hermetically sealed Al pans with the polycrystalline material were prepared in a controlled-atmosphere N_2 glovebox. DSC curves did not show any phase transitions between 110 K and melting points of the samples. Agilent Technologies 1260 Infinity Quaternary LC System connected to an Agilent Technologies 6120 Single Quadrupole LC/MS System was used for High Performance Liquid Chromatography (HPLC) analyses. Crystals of mixed solutions were dissolved separately in MeCN before subjected to HPLC.

Synthetic Methods

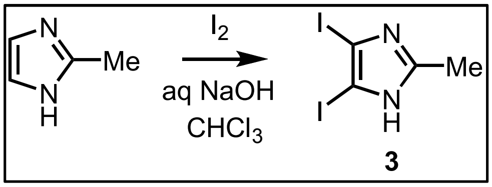

4,5-Diiodo-2-methylimidazole (3): Iodination of 2-methylimidazole was performed following the synthetic procedure reported[16] for the preparation of 2-ethyl-4,5-diiodoimidazole. A solution of 2-methylimidazole (1.24 g, 15.1 mmol) in 2M aqueous NaOH (32 mL) was added to a solution of iodine (7.67 g, 30.2 mmol) in $CHCl_3$ (37 mL). The mixture was stirred at room temperature for 26 h. A saturated aqueous $Na_2S_2O_3$ solution (40 mL) was added to the reaction mixture and the $CHCl_3$ layer was discarded. Neutralisation of the aqueous layer to pH 7 by adding AcOH precipitated a crude yellow product which was collected by filtration and washed with H_2O (40 mL). Recrystallisation from H_2O, after treatment with activated charcoal, gave pure 4,5-diiodo-2-methylimidazole (**3**) as a white powder (4.42 g, 88%). ^{1}H NMR (CD_3SOCD_3, 298 K, 500 MHz): δ 12.48 (s, 1H), 2.27 (s, 3H). ^{13}C NMR (CD_3SOCD_3, 298 K, 126 MHz): δ 150.0, 94.4, 76.0, 13.9. HRMS: (*m*/*z*) calcd for $[M + H]^+$: 334.8537; found 334.8535.

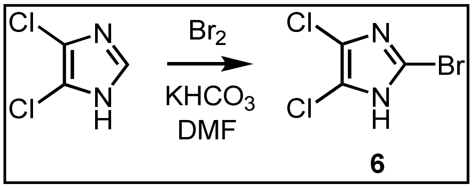

2-Bromo-4,5-dichloroimidazole (6): 4,5-Dichloroimidazole was prepared following an established literature procedure[10]. Neat Br_2 (2.42 g, 14.6 mmol) was added dropwise with stirring to a mixture of 4,5-dichloroimidazole (2.01 g, 14.6 mmol), $KHCO_3$ (1.46 g, 14.6 mmol) and DMF (10 mL) at 0 °C. The reaction mixture was heated to 95 C and stirred at that temperature for 3 h until complete as indicated by TLC (CH_2Cl_2/MeOH 10/1, R_f = 0.46). After cooling, H_2O (100 mL) was added, and the precipitate was collected by filtration, washed with H_2O (30 mL), dissolved in 5% aqueous NaOH and precipitated by adding 10% aqueous HCl. The solid was collected by filtration and washed with copious amounts of H_2O until the filtrate was neutral. Drying afforded 693 mg (22%) of 2-bromo-4,5-dichloroimidazole (**6**) as a white solid. ^{1}H NMR ($CDCl_3$, 298 K, 500 MHz): δ 9.18 (s, 1H). ^{13}C NMR (CD_3COCD_3, 298 K, 126 MHz): δ 128.8, 119.8, 114.1. HRMS: (*m*/*z*) calcd for $[M + H]^+$: 216.8749; found 216.8746.

2,4-Dibromo-5-chloroimidazole (7): The synthesis of 2,4-dibromo-5-chloroimidazole was carried out in two steps starting from 4-bromoimidazole. 4-Bromo-5-chloroimidazole was synthesised following the previously reported[10] literature procedure. Neat Br_2 (838 mg, 5.2 mmol) was added dropwise with stirring to a mixture of 4-bromo-5-chloroimidazole (930 mg, 5.1 mmol), $KHCO_3$ (518 mg, 5.2 mmol) and DMF (10 mL) at 0 °C. The reaction mixture was heated to 95°C and stirred at that temperature for 3 h until complete as indicated by TLC (CH_2Cl_2/MeOH 10/1, $R_f = 0.56$). After cooling, H_2O (100 mL) was added, and the precipitate was collected by filtration, washed with H_2O (30 mL), dissolved in 5% aqueous NaOH, and precipitated by adding 10% aqueous HCl. The solid, which was collected by filtration and washed with copious amounts of H_2O until the filtrate was neutral, was dried, yielding 900 mg (67%) of 2,4-dibromo-5-chloroimidazole (**7**) as a white crystalline powder. ^{1}H NMR ($CDCl_3$, 298 K, 500 MHz): δ 9.21 (s, 1H). ^{13}C NMR (CD_3COCD_3, 298 K, 126 MHz): δ 125.7, 115.6, 103.0. HRMS: (*m*/*z*) calcd for $[M + H]^+$: 260.8246; found 260.8254.

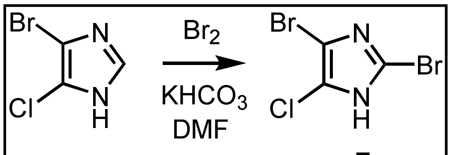

7

4,5-Dichloro-2-ethylimidazole (9): 2-Ethylimidazole (1.00 g, 10 mmol) was added to a solution of NaOH (400 mg, 10 mmol) in 16.7 g of 10–15 % aqueous NaOCl with stirring. The colour of the solution transitioned from its initial colourless state through yellow to deep orange during a 5 min reaction period. The pH was adjusted to 4 with conc HCl, resulting in the precipitation of a crude orange coloured product. The precipitate was collected by filtration, washed with H_2O and dried. Recrystallisation from H_2O, after treatment with activated charcoal, gave pure 4,5-dichloro-2-ethylimidazole (**9**) as a white solid (446 mg, 27%). ^{1}H NMR ($CDCl_3$, 298 K, 500 MHz): δ 9.33 (s, 1H), 2.70 (q, $J = 7.6$ Hz, 2H), 1.30 (t, $J = 7.6$ Hz, 3H). ^{13}C NMR ($CDCl_3$, 298 K, 126 MHz): δ 147.44, 124.95, 109.52, 22.45, 12.15. HRMS: (*m*/*z*) calcd for $[M + H]^+$: 164.9981; found 164.9985.

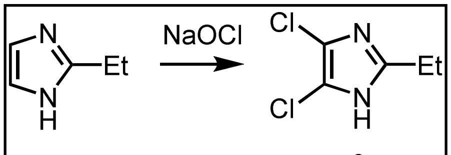

9

Supplementary References

1 Serpell, C. J. & Beer, P. D. Intermolecular interactions in bromo-, methyl-, and cyanoimidazole derivatives. *Cryst. Growth Des.* **13**, 2866–2871, (2013).

2 Andrzejewski, M., Marciniak, J., Rajewski, K. W. & Katrusiak, A. Halogen and hydrogen bond architectures in switchable chains of di- and trihaloimidazoles. *Cryst. Growth Des.* **15**, 1658–1665, (2015).
3 Popelier, P. L. A. Characterization of a dihydrogen bond on the basis of the electron density. *J. Phys. Chem. A* **102**, 1873–1878, (1998).
4 Owczarek, M., Majerz, I. & Jakubas, R. Weak hydrogen and dihydrogen bonds instead of strong N–H···O bonds of a tricyclic [1,2,4,5]-tetrazine derivative. Single-crystal X-ray diffraction, theoretical calculations and Hirshfeld surface analysis. *CrystEngComm* **16**, 7638–7648, (2014).
5 Koch, U. & Popelier, P. L. A. Characterization of C–H···O hydrogen bonds on the basis of the charge density. *J. Phys. Chem.* **99**, 9747–9754, (1995).
6 Mata, I., Alkorta, I., Espinosa, E. & Molins, E. Relationships between interaction energy, intermolecular distance and electron density properties in hydrogen bonded complexes under external electric fields. *Chem. Phys. Lett.* **507**, 185–189, (2011).
7 Bader, R. F. W., Slee, T. S., Cremer, D. & Kraka, E. Description of conjugation and hyperconjugation in terms of electron distributions. *J. Am. Chem. Soc.* **105**, 5061–5068, (1983).
8 Dey, D., Mohan, T. P., Vishalakshi, B. & Chopra, D. Computational study of the formation of short centrosymmetric N–H···S supramolecular synthon and related weak interactions in crystalline 1,2,4-triazoles. *Cryst. Growth Des.* **14**, 5881–5896, (2014).
9 Jungk, T., Hoffmann, Á. & Soergel, E. Challenges for the determination of piezoelectric constants with piezoresponse force microscopy. *Appl. Phys. Lett.* **91**, 253511, (2007).
10 Lutz, A. W. & Delorenzo, S. Novel halogenated imidazoles. Chloroimidazoles. *J. Heterocycl. Chem.* **4**, 399–402, (1967).
11 Bahnous, M., Mouats, C., Fort, Y. & Gros, P. C. Convenient multi-gram scale synthesis of polybrominated imidazoles building blocks. *Tetrahedron Lett.* **47**, 1949–1951, (2006).
12 Grosse, S. *et al.* Access to imidazo[1,2-*a*]imidazolin-2-ones and functionalization through Suzuki–Miyaura cross-coupling reactions. *Eur. J. Org. Chem.* **2013**, 4146–4155, (2013).
13 Gilligan, P. J. & Bakthavatchalam, R. Synthesis of 6-substituted imidazo[4,5-*d*]pyridazin-7-ones. *Heterocycles* **60**, 1329–1337, (2003).
14 Iddon, B. & Lim, B. L. Metal-halogen exchange reactions of mono- and poly-halogenoimidazoles. *J. Chem. Soc., Perkin Trans. 1*, 735–739, (1983).
15 Serpell, C. J., Kilah, N. L., Costa, P. J., Félix, V. & Beer, P. D. Halogen bond anion templated assembly of an imidazolium pseudorotaxane. *Angew. Chem. Int. Ed.* **49**, 5322–5326, (2010).
16 Murata, T., Morita, Y., Nishimura, Y. & Nakasuji, K. Hydrogen-bonded networks of 2,2′-substituted 4,4′-biimidazoles: New ligands for the assembled metal complexes. *Polyhedron* **24**, 2625–2631, (2005).
17 Sheldrick, G. A short history of SHELX. *Acta Crystallogr. Sect. A: Found. Crystallogr.* **64**, 112–122, (2008).
18 Dolomanov, O. V., Bourhis, L. J., Gildea, R. J., Howard, J. A. K. & Puschmann, H. OLEX2: a complete structure solution, refinement and analysis program. *J. Appl. Crystallogr.* **42**, 339–341, (2009).
